# Investigating chitin deacetylation and chitosan hydrolysis during vegetative growth in Magnaporthe oryzae

**DOI:** 10.1111/cmi.12743

**Published:** 2017-04-26

**Authors:** Ivey A. Geoghegan, Sarah J. Gurr

**Affiliations:** ^1^ Department of Plant Sciences University of Oxford Oxford UK; ^2^ Geoffrey Pope Building, Biosciences University of Exeter Exeter UK

## Abstract

Chitin deacetylation results in the formation of chitosan, a polymer of β1,4‐linked glucosamine. Chitosan is known to have important functions in the cell walls of a number of fungal species, but its role during hyphal growth has not yet been investigated. In this study, we have characterized the role of chitin deacetylation during vegetative hyphal growth in the filamentous phytopathogen *Magnaporthe oryzae*. We found that chitosan localizes to the septa and lateral cell walls of vegetative hyphae and identified 2 chitin deacetylases expressed during vegetative growth—*CDA1* and *CDA4*. Deletion strains and fluorescent protein fusions demonstrated that *CDA1* is necessary for chitin deacetylation in the septa and lateral cell walls of mature hyphae in colony interiors, whereas *CDA4* deacetylates chitin in the hyphae at colony margins. However, although the Δ*cda1* strain was more resistant to cell wall hydrolysis, growth and pathogenic development were otherwise unaffected in the deletion strains. The role of chitosan hydrolysis was also investigated. A single gene encoding a putative chitosanase (*CSN*) was discovered in *M. oryzae* and found to be expressed during vegetative growth. However, chitosan localization, vegetative growth, and pathogenic development were unaffected in a *CSN* deletion strain, rendering the role of this enzyme unclear.

## 1 INTRODUCTION

Chitin, a polymer of β‐1,4‐linked *N*‐acetylglucosamine, is unique amongst the major polysaccharide components of the fungal cell wall in that it is able to be chemically modified by deacetylation. Chitin deacetylation is catalyzed by a family of carbohydrate esterase enzymes known as chitin deacetylases (CDAs), belonging to the Carbohydrate Esterase 4 family, according to Carbohydrate‐Active EnZymes database (CAZy) classification. The product of this deacetylation is an often heterogenous polymer of glucosamine and *N*‐acetylglucosamine, known as chitosan. CDAs are broadly conserved, known to occur not only in all fungi (Ruiz‐Herrera & Ortiz‐Castellanos, 2010) but also in insects, where they have a number of developmental roles, including in tracheal development and moulting (Arakane et al*.*, 2009; Luschnig, Batz, Armbruster, & Krasnow, 2006; Wang, Jayaram, et al., 2006b; Xi, Pan, Ye, Yu, & Zhang, 2014). Considering the relative chemical and physical properties of chitin and chitosan, we hypothesized that chitin deacetylation could also have key roles in fungal development. Chitin forms highly crystalline, rigid microfibrils in the cell wall (Rinaudo, 2006), but its deacetylation results in the creation of primary amine groups, with a pKa of ~6.5 (Wang, Chen, et al., 2006a). As a result, chitosan is polycationic at physiological pH and therefore soluble and potentially more flexible than chitin. Here, comparisons could be made with the demethylesterification of homogalacturonan (HG) in plant cell walls by pectin methylesterase enzymes. Such demethylesterification has a critical role in the regulation of the mechanical properties of the cell wall and therefore in plant development (reviewed in (Wolf, Mouille, & Pelloux, 2009). HG is secreted in a methylesterified form, only to be demethylesterified *in muro* by pectin methylesterase enzymes, the effects of which are twofold. First, demethylesterification creates anionic carboxyl groups that can be cross‐linked by Ca^2+^, which increases wall rigidity. On the other hand, demethylesterified HG is also more susceptible to hydrolysing enzymes such as endopolygalacturonase (Wolf et al*.*, 2009). Determining whether chitin deacetylation has analogous roles in fungal cell walls could therefore be a valuable line of investigation. It may yield novel insights into the relationship between cell wall composition, the physical properties of the cell wall, and cellular morphogenesis.

Fungal CDAs have currently only been characterized in a very limited number of species. In Saccharomyces cerevisiae and Cryptococcus neoformans, chitosan appears to have a structural role in ascospores and vegetative cells, respectively (Baker, Specht, Donlin, & Lodge, 2007; Christodoulidou, Bouriotis, & Thireos, 1996). Chitosan is also a component of the spore wall in *Ashbya gossypii*. In this case, however, deletion of the sole CDA in this species resulted in a complete loss of sporulation (Lickfeld & Schmitz, 2012), suggesting a possible developmental role for chitin deacetylation. Most recently, we discovered a novel role for chitosan in appressorium development in *Magnaporthe oryzae*. Here, chitosan does not play a morphogenic or structural role but is required for germling adhesion and surface perception (Geoghegan & Gurr, 2016). Taken together, these data suggest that chitosan may have multiple, discrete roles, depending on the species and cell type in question. It is therefore of interest to examine the possible involvement of chitin deacetylation in other developmental processes. Indeed, the role of chitin deacetylation has not hitherto been investigated in hyphal growth. The process of filamentous hyphal growth in fungi involves several morphological changes, including hyphal branching and hyphal tip extension, which could require localized alterations in cell wall flexibility. We hypothesized that these changes to the cell wall could involve the deacetylation of chitin. In order to test this hypothesis, we investigated the role of chitin deacetylation during hyphal growth in *M. oryzae*. In addition, we also examined the extent to which the hydrolysis of chitosan is also involved in cell wall remodelling in *M. oryzae*. There is currently very little functional data regarding the role of chitosanases in fungi. Deletion of the chitosanase CsnB in *Aspergillus fumigatus* had no effect on vegetative growth or sporulation, although it was required for growth on media containing chitosan as a carbon source (Beck, Broniszewska, Schwienbacher, & Ebel, 2014). Similarly, silencing of *CSN1* in *Fusarium solani* also had no effect on mycelial growth or sporulation, although reduced chitosanase activity was recorded (Liu, Zhang, Li, & Bao, 2010). However, because chitin deacetylation has not been investigated in either of these fungi, these studies lack any frame of reference. Indeed, it is difficult to determine the role of a chitosanase without first having prior knowledge of when and where chitosan is synthesized during the life cycle of the fungus. Thus, we sought a more complete understanding of synthesis and turnover in fungi by coupling the characterization of chitosan during vegetative growth with the recently described role of chitin deacetylation during appressorium development in *M. oryzae* (Geoghegan & Gurr, 2016).

## 2 RESULTS

### 2.1 Chitosan is a component of the cell wall in vegetative hyphae

**Figure 1:**
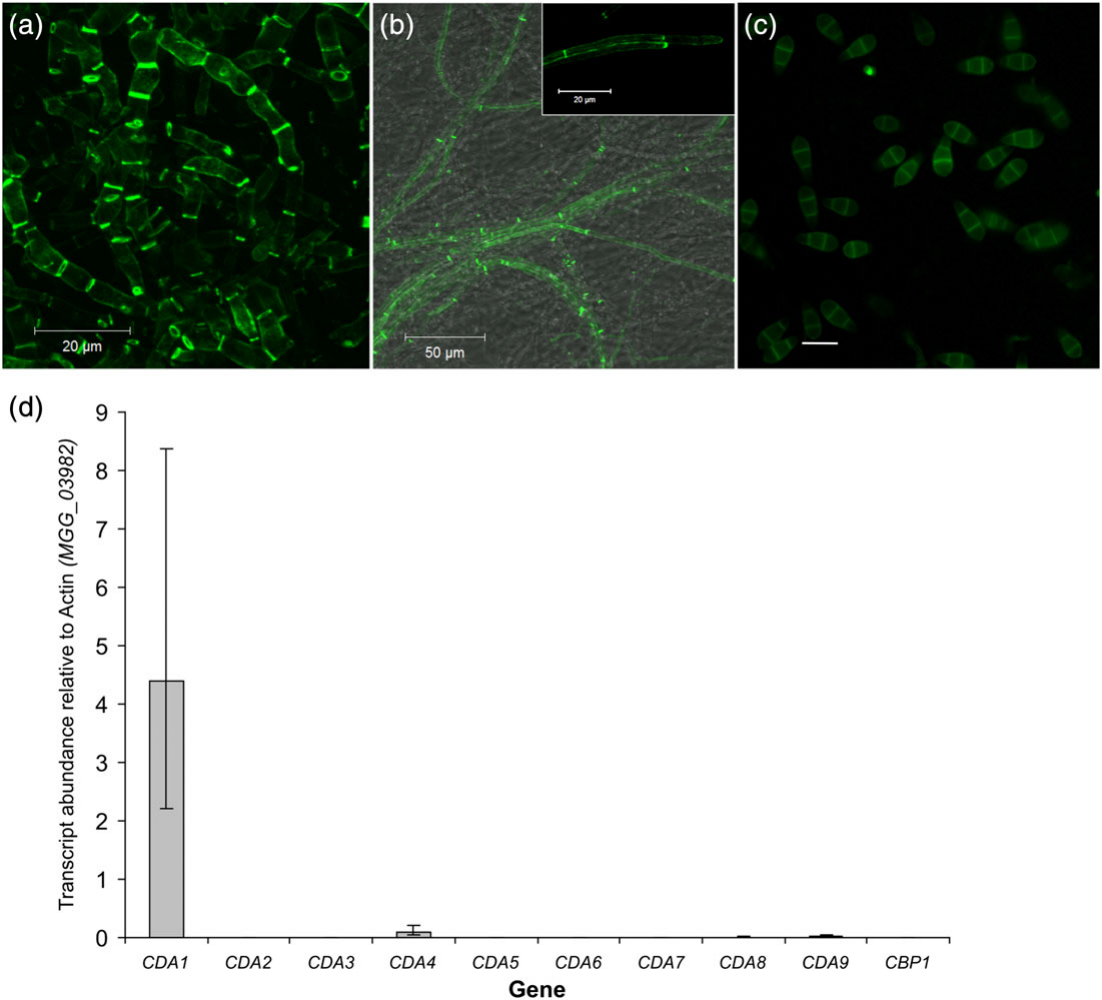
Chitosan is a component of the cell wall in the vegetative hyphae of *Magnaporthe oryzae*. The chitosan specific probe OGA488 (a) was used to test for the presence of chitosan in hyphae grown in liquid complete medium. Strong labelling of septa and lateral wall was observed. (b) OGA488 labelling of hyphae grown on solid complete medium, again showing labelling of septa and lateral walls, but also of hyphal tips (inset). (c) Conidia could be labelled with the fluorescent dye eosin Y, but not OGA488 (not shown), scale bar: 20 μm. (d) Expression of the chitin deacetylases during vegetative growth in liquid complete medium. Expression levels of the CDAs were analyzed by qRT‐PCR and normalized to actin (MGG_03982). Error bars show standard deviation, *n* = 3. CDA = chitin deacetylase

In order to first determine whether chitosan is a component of the cell wall in the vegetative hyphae of *M. oryzae*, two approaches were used. First, mycelium resulting from growth in liquid complete medium (CM) was stained with the anti‐chitosan antibody mAbG7 (Schubert et al*.*, 2010). However, this revealed only weak staining of the hyphae (Figure S1), which nevertheless suggested the presence of chitosan in the cell walls of vegetative hyphae. In the second approach, mycelium was stained with the recently developed chitosan‐specific fluorescent probe OGA488 (Mravec et al*.*, 2014). In this case, much stronger staining of hyphae was observed (Figure 1a), which is perhaps attributable to the superior penetrability of the OGA488 probe compared with the antibody. Septa in particular demonstrated very strong fluorescence. Intriguingly, this fluorescence was often restricted to the outer edge of the septa, thereby giving rise to an “annular” pattern of fluorescence. Unfortunately, the organization and distribution of hyphae are not easily apparent upon microscopic observation of mycelial pellets, making it difficult to draw any conclusions about the developmental regulation of chitin deacetylation during vegetative growth. In an attempt to shed light on this question, staining with OGA488 was also performed of hyphae, which had been grown over a glass coverslip, which had previously been overlaid with solid CM. In this case, staining was not observed in hyphae growing at the outermost edge of the colony but was typically first observed 1.5–2 mm from this point. Here, strong labelling of septa was again observed, as well as weaker labelling of lateral walls, and occasionally of hyphal tips (Figure 1b). However, the extreme hydrophobicity of hyphae grown on solid medium prevented the labelling of all but the outer ~5 mm of the colony, as washing buffer and OGA488 failed to penetrate beyond the surface hyphae.

Chitosan is known to be a component of the cell wall in the ascospores of *A. gossypii* (Lickfeld & Schmitz, 2012) and *S. cerevisiae* (Christodoulidou, Briza, Ellinger, & Bouriotis, 1999; Christodoulidou et al*.*, 1996). To determine whether this was also the case in the conidia of *M. oryzae*, staining was performed with both eosin Y and OGA488. Although no staining was observed with OGA488 (not shown), weak fluorescence was observed in the cell wall of conidia labelled with eosin Y (Figure 1c). Considering the highly specific nature of the interaction between OGA488 and chitosan (Mravec et al*.*, 2014), this suggests that chitosan may not be a component of the cell wall in conidia. The weak staining observed with eosin Y may be a result of a nonspecific electrostatic interaction with another cationic cell wall component.

### 2.2 Deletion of CDAs operating during vegetative growth

Having established that chitin is deacetylated during hyphal growth, the next objective was to determine which CDAs were responsible. The genome of *M. oryzae* was previously found to contain 10 putative *CDA* genes (Geoghegan & Gurr, 2016). In order to find which of these genes were expressed during vegetative growth, we performed quantitative reverse transcription polymerase chain reaction analysis on RNA extracted from mycelia grown in liquid CM. Relative transcript abundances for all 10 *CDA* genes revealed that *CDA1* was by far the most highly expressed CDA in this tissue, followed by *CDA4* (Figure 1d). These data align well with a published transcriptomics dataset (Soanes, Chakrabarti, Paszkiewicz, Dawe, & Talbot, 2012).

From the transcriptional analysis outlined above, it was clear that two *CDA* genes were responsible for chitin deacetylation during vegetative growth: *CDA1* and *CDA4*.

Cda1 is a predicted secreted protein with a polysaccharide deacetylase domain (Pfam 01522) and a C‐terminal chitin binding domain (Pfam 00187; Figure S2). Cda4 is also a predicted secreted protein, with a polysaccharide deacetylase domain (Pfam 01522), but also a putative transmembrane domain at its C‐terminus (Figure S2). In order to characterize the role of chitin deacetylation by these two proteins, targeted deletion of *CDA1* and *CDA4* was performed. In addition, *CDA5* was also deleted, both alone and in combination with *CDA4*. Like Cda4, Cda5 also has a putative single transmembrane domain at its C‐terminus, and so it was considered likely that it may demonstrate redundancy with Cda4. Successful replacement of the target genes by antibiotic resistance markers was confirmed by polymerase chain reaction (PCR) and Southern blotting (Figures S3 and S4).

### 2.3 Cell wall composition is altered in the CDA deletion strains

**Figure 2:**
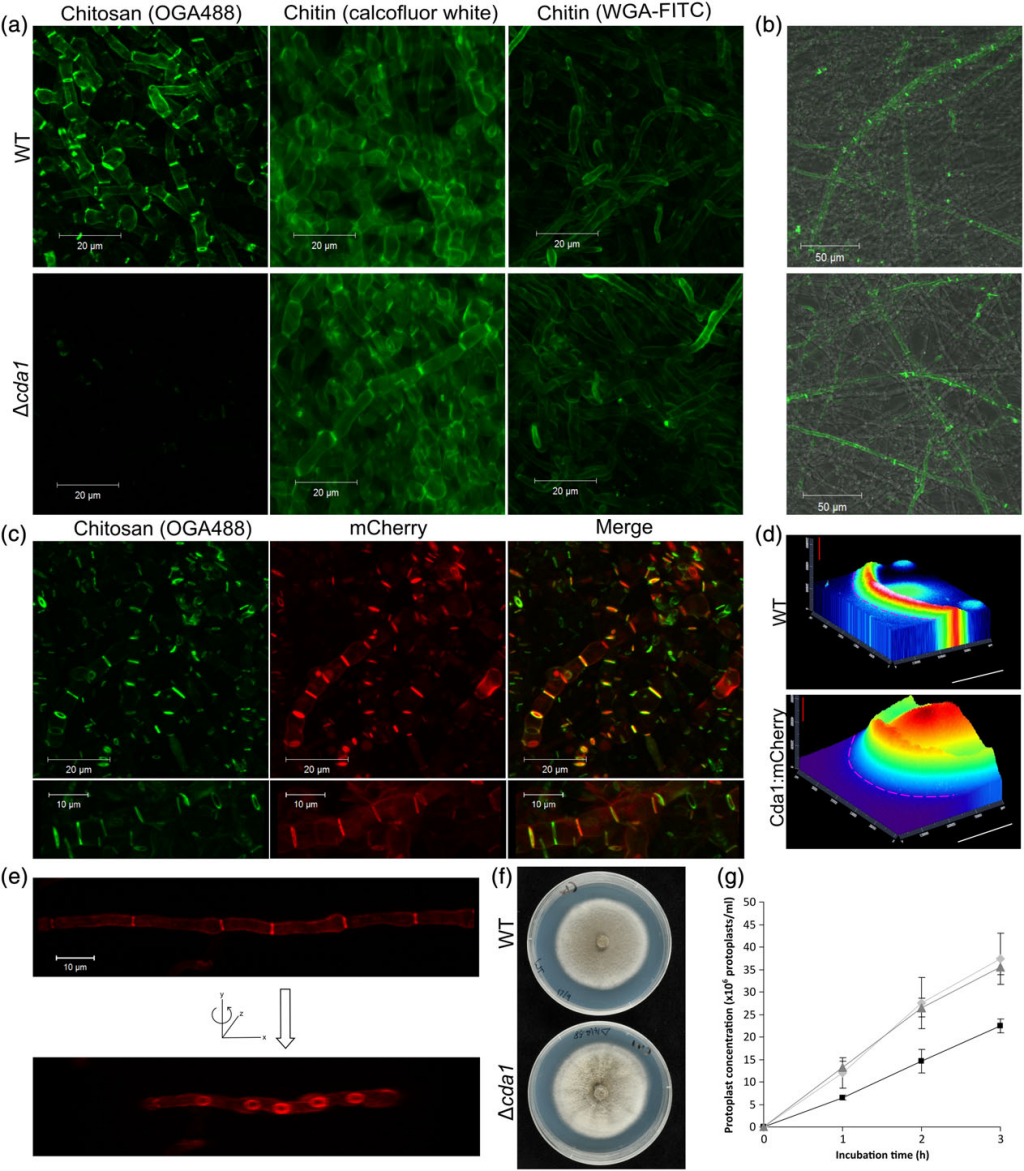
Cda1 is required for chitin deacetylation in mature hyphae. (a) Mycelial growth after a 72‐hr incubation in liquid complete medium, stained with OGA488, WGA‐FITC, or Calcofluor white to test for presence and localization of chitosan and chitin, respectively. Staining with OGA488 was dramatically reduced in the Δcda1 mutant, suggesting a reduction in chitosan, whereas chitin content appeared to be unaffected. Scale bars: 20 μm. (b) Staining of hyphae grown on solid medium (approximately 1.5–2 mm from the colony edge) with OGA488, showing similar labelling in the WT and Δcda1 strains. Scale bars: 50 μm. (c) Complementation of the Δcda1 strain by mCherry‐tagged Cda1. Staining with OGA488 was restored in the complemented strain and colocalized with mCherry fluorescence. Scale bars: 20 μm. (d) Fluorescence heat map of a colony expressing Cda1:mCherry. Fluorescence was not detected at the colony margin (marked by the pink dashed line) but increased strongly towards the colony centre. A small amount of autofluorescence was observed in WT colonies. White scale bars: 10 mm, red scale bars: 20,000 fluorescence units. (e) Maximum projection image of Cda1:mCherry localizing to hyphal septa, showing annular localization (image rotated around y axis). Scale bar: 10 μm. (f) Colonies of WT and Δcda1 strains, growing on complete medium. (g) The Δcda1 mutant is more resistant to cell wall degrading enzymes. Mycelium of the WT (light grey lines with diamonds), Δcda1 (black lines with squares), or Δcda1/CDA1:mCherry (dark grey lines with triangles) strains were incubated with cell wall lysing enzymes. The number of protoplasts released was measured at 1, 2, and 3 hr. Error bars show standard deviation, *n* = 3. WT = wild type; WGA‐FITC = FITC‐labelled wheat germ agglutinin; CDA = chitin deacetylase

**Figure 3:**
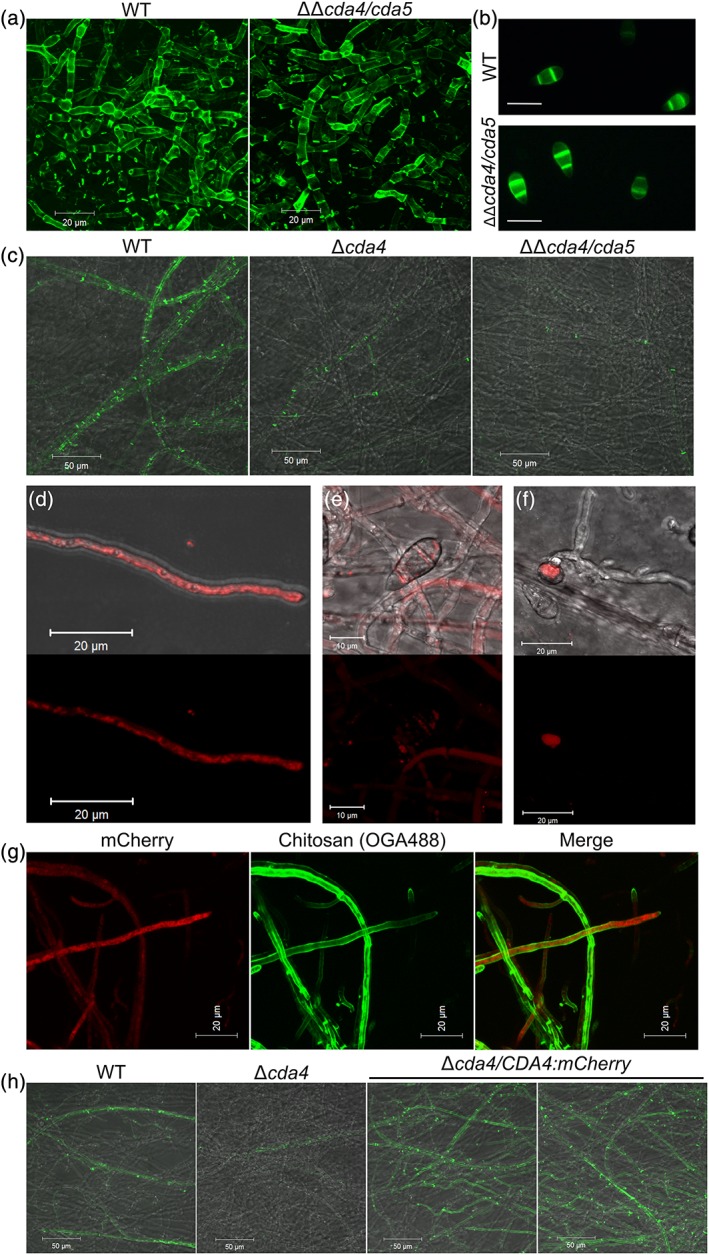
CDA4 and CDA5 are required for chitin deacetylation in vegetative hyphae at the colony periphery. (a) Staining of chitosan in vegetative hyphae grown in liquid media, showing similar staining in the WT and cda4/cda5 strains. (b) Staining of conidia with eosin Y. (c) Staining of chitosan in vegetative hyphae grown on solid media in the WT, Δcda4, and ΔΔcda4/cda5 strains, showing reduced chitosan staining in the deletion strains. Pictures were taken approximately 1.5–2 mm from the colony edge. Scale bars: 20 μm in a and b, 50 μM in c. (d, e, & f). Intracellular localization of Cda4:mCherry in vegetative hyphae, conidia, and appressoria, respectively. Scale bars: 20 μm in d and f, 10 μm in e. (g) Cda4:mCherry does not colocalize with OGA488‐labelled chitosan in vegetative hyphae. Scale bars: 20 μm. (h) Staining of chitosan in vegetative hyphae grown on solid media, showing restored chitosan synthesis in Δcda4/CDA4:mCherry strains (two independent transformant lines are shown). Pictures were taken 1.5–2 mm from the colony edge. Scale bars: 50 μm. WT = wild type; CDA = chitin deacetylase

Following successful gene replacement as described above, chitin deacetylation in the resulting deletion strains was characterized. First, mycelial pellets resulting from 72‐hr growth in CM were stained with OGA488. Although hyphae of the wild‐type (WT) strain demonstrated strong labelling of septa and lateral walls as discussed previously (Figure 1a), labelling was almost absent in Δ*cda1* (Figure 2a). Conversely, labelling in the ΔΔ*cda4*/*cda5* strain was similar to WT (Figure 3a). Mycelial pellets of Δ*cda1* were also stained with both FITC‐labelled wheat germ agglutinin and Calcofluor white (CFW), to test for cell wall chitin content. No clear changes were observed between the hyphae of the WT and Δ*cda1* strains, suggesting that the reduction in chitin deacetylation had not resulted in a concomitant increase in chitin content in Δ*cda1*. It was also apparent from the CFW staining that septum and hyphal morphology were unchanged in the Δ*cda1* strain. Hyphae grown on solid medium were also stained with OGA488. In this case, a dramatic reduction in labelling of hyphae 1.5–2 mm from the colony margins was observed in the Δ*cda4* and ΔΔ*cda4*/*cda5* strains (Figure 3c), whereas staining of these hyphae was unaffected in Δ*cda1* (Figure 2b). Staining was not entirely abolished in ΔΔ*cda4*/*cda5*; septa in particular continued to show some labelling, perhaps due to the action of Cda1. Nevertheless, these data suggest that Cda1 and Cda4 appear to have developmentally distinct roles: Cda1 is responsible for chitin deacetylation in mature hyphae, whereas Cda4 (and perhaps to a lesser extent Cda5) deacetylates chitin in hyphae at colony margins. Conidia of the deletion strains were also stained with eosin Y. However, no change in labelling was observed in either deletion strain (Figure 3b and not shown), nor was there any clear change in conidiation (not shown).

### 2.4 Localization of Cda1 and Cda4

Cda1 and Cda4 appear to have distinct roles in chitin deacetylation during vegetative growth. In order to further examine the roles of these enzymes, the respective deletion strains were complemented by expressing mCherry‐tagged Cda1 or Cda4.

In strains expressing *CDA1:mCherry*, weak fluorescence was observed in the lateral walls of hyphae, with much stronger fluorescence associated with septa. Here, fluorescence localized either in an annular fashion at the outer edge of the septum or to the entire septum (Figure 2c,e), consistent with the pattern of chitosan staining observed with OGA488. Importantly, expression of *CDA1:mCherry* in the deletion strain was able to restore chitin deacetylation; mycelial pellets of the Δ*cda1*/*CDA1:mCherry* strain stained with OGA488 showed strong labelling, which often colocalized with fluorescence of the Cda1:mCherry (Figure 2c), thus proving the functionality of the Cda1:mCherry fusion protein. Notably, colocalization of Cda1:mCherry and OGA488 staining was not complete; in many cases, septa showed OGA488 fluorescence but not Cda1:mCherry fluorescence. These are presumably sites where deacetylation has already taken place, and the Cda1:mCherry protein has become degraded/denatured. Expression of Cda1:mCherry appeared to be restricted to mature hyphae; on solid medium, fluorescence was not observed in hyphae growing at the colony edge. No differences in fluorescence localization were observed between hyphae grown in liquid or on solid medium. Cda1:mCherry fluorescence was also not observed at any other stages in the life cycle, including during appressorium development, conidiation, or *in planta* growth, suggesting that it is specific to the vegetative growth phase. In order to gain further insight into Cda1:mCherry expression at the colony level, colonies expressing Cda1:mCherry were observed under a fluorescence stereoscope. Fluorescence was not detected from the outer ~5 mm of colonies, consistent with observations made with confocal microscopy, but was extremely strong in the entire colony interior (Figure 2d). These findings are consistent with those showing that Cda1 is not required for chitin deacetylation in hyphae at the colony margins, only in mature hyphae in the colony interior.

C‐terminal mCherry‐tagged Cda4 demonstrated an entirely different localization to that described above for Cda1:mCherry. In strains expressing *CDA4:mCherry*, fluorescence was observed in vegetative hyphae near the colony edges (Figure 3d), as expected from the loss of chitosan observed in these hyphae in the Δ*cda4* strain. Fluorescence was also observed in developing conidia (Figure 3e) and in appressoria postpenetration (Figure 3f), but not during appressorium development or in invasive hyphae. Surprisingly however, this fluorescence was entirely intracellular, as evidenced by a lack of colocalization with OGA488 (Figure 3g). In regions of the hyphae more distal to the tip, fluorescence was present in large intracellular bodies characteristic of vacuoles. At hyphal tips, Cda4:mCherry was not observed in the Spitzenkörper but localized to tubular structures, small punctae, or in a more diffuse pattern. Due to the presence of a transmembrane domain at the C‐terminus of Cda4, it was considered that the fusion of mCherry immediately adjacent to this (albeit with a 10aa linker sequence) may have disrupted localization of the protein. In order to independently verify the localization of Cda4 observed with the C‐terminal fusion, an additional mCherry fusion was constructed. In this case, the mCherry was cloned into *CDA4* after the putative signal peptide, such that the mCherry would be at the N‐terminus of the mature protein. However, although strains transformed with this construct exhibited weak fluorescence in comparison to the C‐terminal fusion described above, the localization was similar (Figure S6).

Considering the unexpected localization of Cda4:mCherry, it was important to determine whether the C‐terminal Cda4:mCherry fusion was functional. To test this, hyphae of the complemented strains were grown on solid CM and stained with OGA488. This revealed that chitin deacetylation was fully restored in Δ*cda4* strains expressing *CDA4:mCherry* (Figure 3h). In fact, staining appeared stronger than in the WT strain, suggesting overcomplementation. This, nevertheless, proves the functionality of the Cda4:mCherry fusion protein, although its level of expression or posttranslational regulation may be altered when compared with that of the native protein.

The localization of Cda4:mCherry to small punctae near the hyphal tip was intriguing and reminiscent of the localization of fluorescently tagged chitin synthases observed in numerous previous studies (Fajardo‐Someraa'l et al*.*, 2015; Sanchez‐Leon et al*.*, 2011; Takeshita, Yamashita, Ohta, & Horiuchi, 2006; Weber, Assmann, Thines, & Steinberg, 2006). We hypothesized that Cda4:mCherry may therefore colocalize with a chitin synthase, which would provide a valuable insight into possible coordinated transport of these proteins and the mechanism of chitin deacetylation. There are seven chitin synthases in *M. oryzae*, which could possibly colocalize with Cda4:mCherry (Kong et al*.*, 2012). However, taking into consideration previous work, Chs1 appeared to be a likely candidate; Chs1:eGFP had already been found to localize to hyphal tips and to developing conidia (Kong et al*.*, 2012). To see if Chs1:eGFP colocalizes with Chs4:mCherry, first, the Chs1:eGFP construct was cloned in precisely the same way as described previously (Kong et al*.*, 2012) and transformed into strains expressing *CDA4:mCherry.* However, although Chs1:eGFP and Cda4:mCherry were often expressed in the same hyphae, colocalization of the fusion proteins was not observed. Chs1:eGFP fluorescence localized to the cell periphery and Spitzenkörper at hyphal tips, in contrast to Cda4:mCherry (Figure S7). In addition, although Chs1:eGFP also demonstrated intracellular localization, this only colocalized with Cda4:mCherry in the larger intracellular bodies, most likely vacuoles.

### 2.5 Vegetative growth and pathogenic development are largely unaffected in the CDA deletion strains

To determine the role of chitin deacetylation during vegetative growth, growth of the deletion strains was evaluated under a range of different stress conditions on solid medium. No significant differences in radial growth were observed between the WT and *Δcda1* strains after 10‐day growth (Student's *t* test, *p* < .05; Figure S5), suggesting that chitin deacetylation by Cda1 is not required for optimal radial growth under the conditions tested. In addition, no consistent differences in colony appearance, conidiation or hyphal morphology were observed between the WT and Δ*cda1* strains (Figures 2f and S5). In order to further investigate the properties of the cell wall in the Δ*cda1* strain, a protoplast release assay was performed. Here, the mycelium resulting from 72‐hr growth in CM was harvested and incubated with cell wall lysing enzymes (a mixture of chitinases, glucanases, and proteases). Under these conditions, the Δ*cda1* released fewer protoplasts than either the WT or complemented strain (Δ*cda1*/*CDA1:mCherry*; Figure 2g), suggesting that the cell wall of the Δ*cda1* strain is more resistant to enzymatic lysis.

Vegetative growth was also largely unaffected in the Δ*cda4* and ΔΔ*cda4*/*cda5* strains (Figure S8), with overall colony appearance, conidiation, and hyphal morphology identical to the WT strain (Figure S8). Growth upon media containing caffeine resulted in the largest growth reductions in the deletion strains, although even here, the reduction was only about 15%. This suggests that chitin deacetylation in hyphae at colony margins is also not required for optimal growth under the conditions tested.

Pathogenic development was also evaluated in the *CDA* deletion strains. Conidia of deletion strains germinated on a hydrophobic glass surface developed appressoria with identical morphology to WT (Figures S5 and S9). Pathogenicity was also unaffected in the *cda* strains; rice leaves inoculated with conidia of the deletion strains demonstrated similar lesion density to the WT strain (Figures S5 and S9). *CDA1*, *CDA4*, and *CDA5* are therefore not required for pathogenic development in *M. oryzae*, which is as expected, considering their apparent specificity to the vegetative growth phase.

### 2.6 Ectopic expression of CDA1:mCherry

The deacetylation of chitin had been hypothesized to be required for increasing cell wall flexibility during morphogenic events. However, the deletion of *CDA1* or CDA4 did not result in any growth or morphological differences. A separate approach was therefore sought, in order to further investigate a possible link between chitin deacetylation and morphogenesis. We therefore decided to ectopically express *CDA1:mCherry*. The *CDA1:mCherry* construct was placed under control of the constitutive *EF1* promoter (Omar, Bentley, Morieri, Preston, & Gurr, 2016) and transformed into the WT strain. Following successful transformation, several independent lines ectopically expressing Cda1:mCherry were identified and characterized during vegetative growth and pathogenic development.

**Figure 4:**
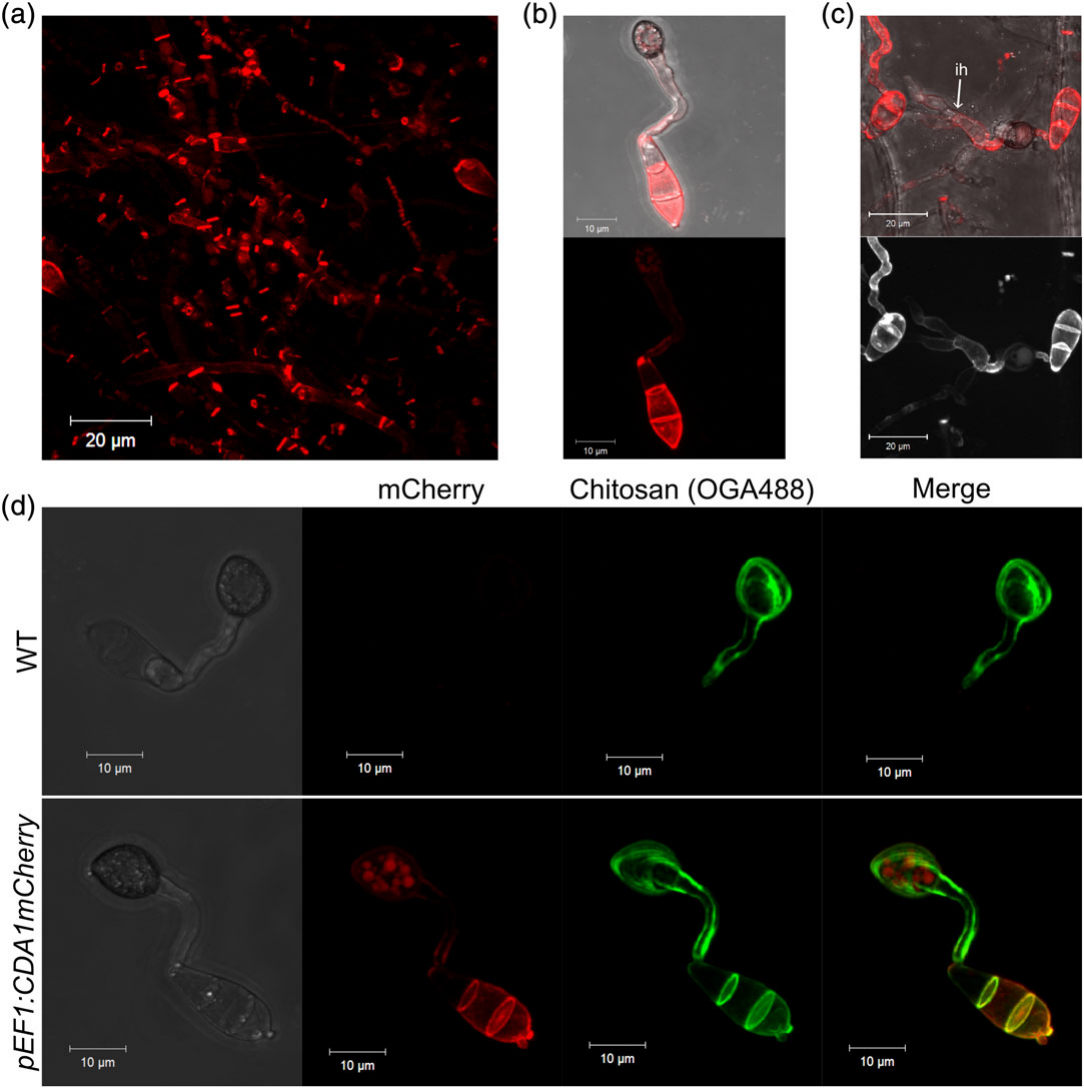
Ectopic expression of CDA1:mCherry results in ectopic chitin deacetylation but no morphological defects. (a) Localization of Cda1:mCherry in vegetative hyphae when expressed by the EF1 promoter, showing similar localization to natively expressed Cda1:mCherry. Localization of Cda1:mCherry in germlings at 24 hpi on an artificial surface (b) and onion epidermis (c) showing fluorescence of conidial cell wall and invasive hyphae (fluorescence pseudocoloured white in bottom panel of c). (d) Localization of Cda1:mCherry at 24 hpi in germlings and colocalization with the chitosan‐specific probe OGA488. Appressorium development was unaffected in pEF1:CDA1:mCherry strains, but ectopic chitin deacetylation was observed in the conidial cell wall. Scale bars: 20 μm in a and c, 10 μm in b and d. WT = wild type; CDA = chitin deacetylase

During vegetative growth, strong fluorescence localized to the hyphal septa, with weaker fluorescence at the lateral walls (Figure 4a), just as observed previously with the natively expressed Cda1:mCherry. Such fluorescence also appeared to be restricted to the mature hyphae and was not observed in those growing at colony margins. Hyphal morphology was unaffected by ectopic expression of Cda1:mCherry as was overall colony appearance (not shown). In conidia germinated on a hydrophobic glass surface, fluorescence was observed in the cell walls of the conidia, germ tubes, and appressoria, although intracellular localization (most likely vacuolar) was also apparent (Figure 4b). During invasive growth, cell wall‐localized fluorescence was also observed in invasive hyphae (Figure 4c), but morphology was, once again, unaffected. Although germling morphology was identical to the WT in *pEF1:CDA1mCherry* strains, staining with OGA488 demonstrated that ectopic chitin deacetylation had indeed occurred in the conidial wall (Figure 4d).

### 2.7 A single chitosanase is present in M. oryzae

It is clear from the data presented thus far that chitosan is a component of the cell wall during vegetative growth in *M. oryzae*. Because all major polysaccharides in the fungal cell wall have enzymes responsible for their hydrolysis (e.g., chitinases, glucanases, and mannanases), an enzyme responsible for the hydrolysis of chitosan is likely to also be present in *M. oryzae*. It was reasoned that characterizing the role of such an enzyme could yield further insights into the role of chitosan during vegetative growth*.*

In order to determine the number of putative chitosanase genes in the *M. oryzae*, the *A. fumigatus* Csn protein sequence (Genbank AAO41660.1; Cheng, Chang, Wu, & Li, 2006) was used as a Basic Local Alignment Search Tool (BLAST) query against the *M. oryzae* protein database (http://www.broadinstitute.org/annotation/genome/magnaporthe comparative/MultiHome.html). This identified just a single putative chitosanase (MGG07656). Further BLAST searches of the protein database using MGG07656 as a query and Pfam domain searches to find additional proteins with a GH75 domain (Pfam 07335) failed to identify further chitosanase sequences. Therefore, *M. oryzae* has just a single putative chitosanase (Csn) in its genome. The MGG07656 *CSN* gene is 2,579 nt in length, with two introns, and encodes a protein of 797 aa. This is significantly longer than all other chitosanase sequences, which are typically 250–350 aa in length. Closer inspection of the predicted protein sequence reveals that in addition to an N‐terminal signal peptide (SignalP 4.0) and a chitosanase domain (Pfam 07335), the C‐terminal ~450 aa is almost entirely composed of short repeats enriched in proline (P), aspartate (D), and lysine (K) residues, which are predicted to give rise to a highly disordered protein structure (Figure S9). Both the genomic DNA of the MGG_07656 locus and the complementary DNA were sequenced (not shown), confirming the presence of these repeats. Comparison of the *M. oryzae* Csn protein sequence with those of all other Csn in other fungi (Table S2) suggests that the *M. oryzae* chitosanase is unique with regard to these repeat sequences. However, a BLAST search using these repeat sequences as a query revealed a number of proteins with similar types of repeat sequences, including the cell wall protein BibA from Streptococcus agalactiae (Santi et al*.*, 2007), procyclic acidic repeat proteins from *Trypanosoma brucei* (Mowatt & Clayton, 1987), and the periplasmic protein TonB from *Eschericia coli* (Postle & Good, 1983).

Alignment of chitosanase sequences from a number of fungi (Figure S9) shows that the key catalytic residues (aspartate and glutamate), as identified by Cheng et al. (Cheng et al*.*, 2006), are conserved, suggesting that the *M. oryzae* Csn is a functional endo‐chitosanase.

### 2.8 Chitosanases are restricted to the Pezizomycotina

CDAs are present in all fungi (Ruiz‐Herrera & Ortiz‐Castellanos, 2010). To determine whether this is also true of chitosanases, a reciprocal best‐hit BLAST search was performed on the protein databases of all sequenced fungal genomes, using the *M. oryzae* Csn as a query sequence. This revealed that chitosanases are restricted to the Pezizomycotina subphylum (Table S2); no putative chitosanases were identified in any other subphylum of the Ascomycota nor any phylum outside of the Ascomycota. Even within the Pezizomycotina, multiple, independent gene losses have apparently occurred; no chitosanase was identified in *Magnaporthe poae*, neither in the Ajellomycetaceae nor in the Helotiales, for example. In most of those species with putative chitosanases, just a single sequence was present. *Aspergillus spp*. were an exception, with four, as discovered previously (Cheng et al*.*, 2006). Thus, the distribution and number of chitosanases do not mirror that of the CDAs.

### 2.9 Chitosanase is expressed in vegetative hyphae but is not required for normal vegetative growth or development

**Figure 5:**
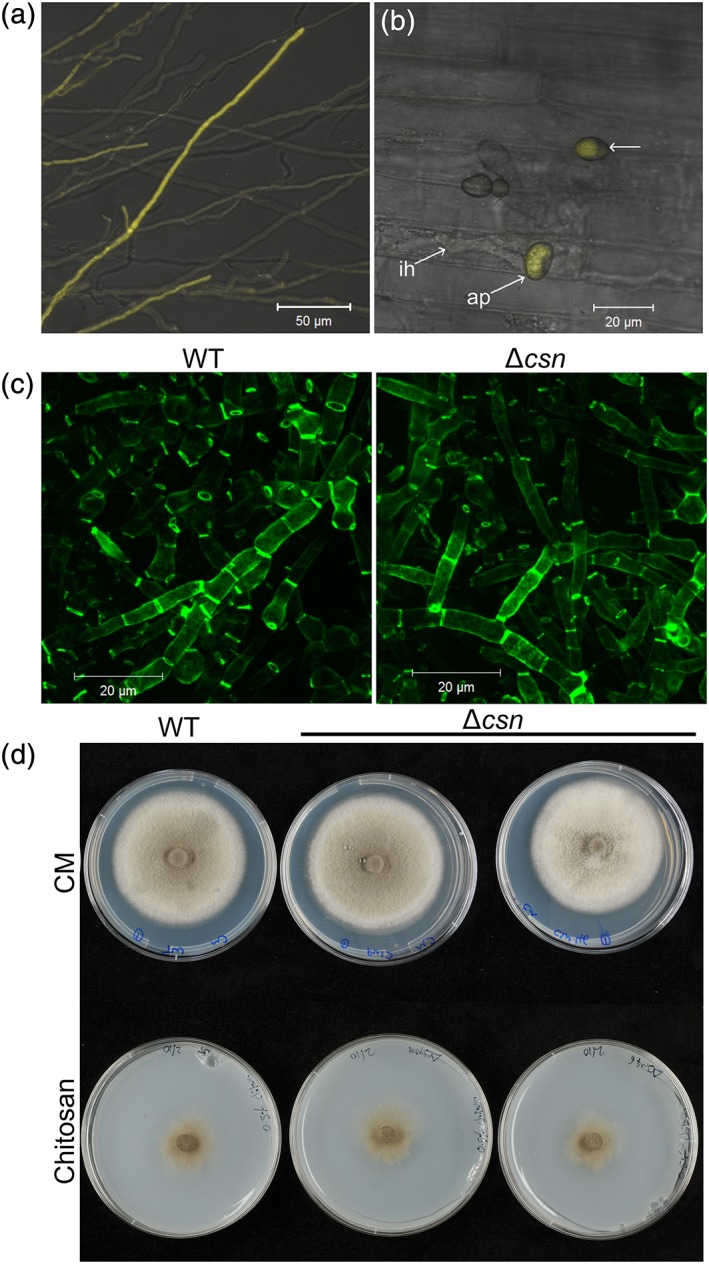
Characterization of the Δcsn strain. (a) Expression of yellow fluorescent protein by pCSN in vegetative hyphae growing on complete medium. Scale bar: 50 μm. (b) CSN promoter activity was not detected in invasive hyphae during *in planta* growth but was detected in appressoria postpenetration. (c) Labelling of chitosan on vegetative hyphae grown in liquid complete medium, using the chitosan‐specific probe OGA488. Similar staining was observed in both WT and Δcsn strains. Scale bars: 20 μm. (d) Growth of Δcsn (two independent lines shown) on either complete medium or medium containing 0.5% chitosan (*w*/*v*) as a sole carbon source, showing similar growth to the WT strain. WT = wild type; CDA = chitin deacetylase; CSN = chitosanase

Chitin deacetylation is known to occur at multiple different stages of the life cycle in *M. oryzae* (Geoghegan & Gurr, 2016). To determine whether the expression of *CSN* is coincident with chitin deacetylation, a promoter fusion was created. The upstream sequence of *CSN* was cloned upstream of three copies of *YFPvenus*, and the entire construct transformed into WT *M. oryzae.* In the resulting transformants, fluorescence was observed most strongly in vegetative hyphae, particularly those at the colony margins (Figure 5a). Additionally, fluorescence was observed in appressoria postpenetration (~48 hpi; Figure 5B), but neither in invasive hyphae nor during appressorium development (not shown). Therefore, the expression of *CSN* is not coincident with chitin deacetylation in *M. oryzae*.

We also attempted to determine the localization of the chitosanase protein, by creating a C‐terminal eGFP fusion. However, although fluorescence was observed in vegetative hyphae, as with the promoter fusion, fluorescence was entirely cytoplasmic (Figure S11). This may suggest mislocalization of the fusion protein because the presence of an N‐terminal signal peptide would normally suggest a cell wall localization.

Based on the promoter analysis, it appears that *CSN* has a role in vegetative growth. In order to characterize this role, a deletion strain for *CSN* was created. The entire coding sequence of *CSN* was replaced with a gene encoding resistance to bialaphos, and successful deletion was confirmed by PCR and Southern blotting (Figure S12). Two independent deletion lines (named 9 and 46) were chosen for characterization.

Δ*csn* strains were first stained for the presence of chitosan, to determine whether deletion of *CSN* had resulted in alterations in either the amount or distribution of chitosan. However, staining of vegetative hyphae with OGA488, germlings with the anti‐chitosan antibody mAbG7 (Schubert et al*.*, 2010), or conidia with eosin Y did not reveal any differences in the patterns or intensity of staining in Δ*csn*, compared with the WT strain (Figures 5b and S13). The morphology of hyphae, germlings, and conidia was also unaffected in the Δ*csn* strain.

Radial growth of the Δ*csn* strain was assessed on solid media under a range of stress conditions. As with the *CDA* deletion strains assessed in this study, radial growth was largely unaffected in the Δ*csn* strain, although some small yet statistically significant changes were detected under certain conditions, including SDS and H_2_O_2_ (Figure S14).

In *A. fumigatus*, deletion of CsnB resulted in reduced growth on media containing chitosan (Beck et al*.*, 2014). To see if this was also true of the Δ*csn* strains in *M. oryzae*, growth assays with chitosan as a carbon source were performed on solid and in liquid medium. On solid medium containing 0.5% chitosan (*w*/*v*), equal growth was observed in all strains after a 14‐day incubation (Figure 5d). In some fungi, a “halo” indicative of substrate degradation is also observed around fungal colonies grown on chitosan (Palma‐Guerrero, Jansson, Salinas, & Lopez‐Llorca, 2008), but this was not observed for *M. oryzae*. In liquid medium, chitosan was clearly able to be used as a carbon source, because biomass was much higher in flasks containing 0.33% chitosan (*w*/*v*; and 16.6 mM sodium acetate) than those containing sodium acetate alone. However, the biomass of the Δ*csn* strains was identical to the WT strain (Figure S13).

Cell wall hydrolases have previously been implicated in autolysis under starvation conditions. To determine whether Csn is required for autolysis, equal concentrations of conidia were inoculated into flasks containing minimal media and incubated for up to 30 days, with biomass measurements taken every 4 days after an initial 5‐day incubation.

Biomass increased up to 17 dpi, then decreased between 21 and 30 dpi, suggesting the onset of autolysis (Figure S13). However, the biomass of the WT and Δ*csn* strains was similar across the duration of the experiment, suggesting that chitosanase is not required for autolysis.

### 2.10 Pathogenicity and pathogenic development are unaffected by deletion of CSN

The cell walls of appressoria and invasive hyphae appear to have a particularly high chitosan content (Geoghegan & Gurr, 2016). In order to determine whether chitosanase is required for morphogenesis in these structures, conidial germination, appressorium development, and pathogenicity were evaluated. Germination and appressorium development on an artificial hydrophobic surface were unaffected in the Δ*csn* mutant at all time points (Figure S14c). Conidia of Δ*csn* inoculated onto barley leaves also resulted in similar lesion densities to the WT (Figure S14d), with identical results obtained on rice leaves (not shown). Morphology of invasive hyphae in onion epidermis was also unaffected in the Δ*csn* mutant (not shown).

## 3 DISCUSSION

Based on the conserved nature of CDAs in fungi, chitosan is presumably a ubiquitous component of the fungal cell wall. Yet there is limited information regarding the occurrence and biological role of chitosan in fungi, particularly with respect to its possible involvement in cellular morphogenesis during vegetative hyphal growth. In this study, chitosan has been found to be a component of the cell wall in the vegetative hyphae of *M. oryzae*, suggesting that chitosan has roles beyond those already described for pathogenic development in this fungus (Geoghegan & Gurr, 2016). Two CDAs (Cda1 and Cda4) were found to be necessary for chitin deacetylation in vegetative hyphae. Interestingly, they appear to be involved in two distinct modes of chitin deacetylation, in discrete zones of colonies.

### 3.1 Chitin deacetylation in mature hyphae

Cda1, a predicted secreted protein with a CDA domain and a single C‐terminal chitin‐binding domain, is required for chitin deacetylation in the cell walls of mature hyphae located in the colony interior. The role of chitin deacetylation in these hyphae is currently unclear. Expression of Cda1 is restricted to mature hyphae, and the mCherry fusion of Cda1 did not demonstrate localization indicative of a role in morphogenesis, for example, localizing to sites of hyphal branching. This suggests that it does not play a direct role in growth or hyphal morphogenesis. This hypothesis is consistent with the fact that radial growth of the Δ*cda1* mutant on solid media was identical to the WT strain under a range of stress conditions, including cell wall perturbants (Congo Red and CFW), detergents (SDS), and hyperosmotic stress. Chitin deacetylation by Cda1 may instead be associated with secondary cell wall development during the process of hyphal maturation, perhaps being coincident with other processes such as melanization (although no defects in melanization were observed in the deletion strains). The mycelium of the Δ*cda1* mutant was less susceptible to degradation by cell wall lysing enzymes, suggesting that the properties of the cell wall were in some way altered by the loss of chitin deacetylation. However, this result is somewhat counterintuitive; one would expect the loss of chitosan to render the cell wall more susceptible to degradation by chitinases, because chitosan is a poor substrate for these enzymes (Hartl, Zach, & Seidl‐Seiboth, 2012). The increased resistance of the cell wall to enzymatic degradation in Δ*cda1* could perhaps be attributed to a reduction in cell wall permeability. Chitin is known to be a highly crystalline polysaccharide and therefore has low permeability. The deacetylation of chitin by Cda1 could cause loss of crystallinity and therefore increased permeability, allowing hydrolytic enzymes better access to substrates in the cell wall. This could relate to the biological role of chitin deacetylation at septa, where both chitosan and Cda1:mCherry were found to localize most strongly. Septa are known to be sites of protein secretion in hyphae (Hayakawa, Ishikawa, Shoji, Nakano, & Kitamoto, 2011), and so efficient secretion from septa may be dependent upon high cell wall permeability here. Such permeability may be imparted by chitin deacetylation by Cda1. Chitosan has also been shown to localize to hyphal septa in *Neurospora crassa* (Mravec et al*.*, 2014), suggesting that this may be a conserved feature of the septal cell wall in filamentous fungi.

An additional role for chitin deacetylation by Cda1 in mature hyphae could be as part of a carbon‐recycling mechanism. The cell wall is a major carbon source, and so its deconstruction could serve to redistribute this carbon from the aged parts of the fungal colony to the growing edge. Chitin deacetylation could either serve as a direct source of carbon in the form of acetate (released by the deacetylation reaction) or be the first step to making the cell wall of old hyphae more susceptible to degradation by endogenous chitinases and glucanases. However, no evidence for a role in carbon recycling was found in the present study. First, Cda1 appears to be highly expressed even after a relatively short incubation in nutrient‐rich CM, at which point carbon recycling from the cell wall would probably not be necessary. Second, under conditions of carbon or nitrogen starvation on solid medium, no reduction in radial growth was observed. Yet further investigation is required before this hypothesis can be dismissed; more sensitive methods may be required to study the efficiency of carbon recycling from the cell wall in the Δ*cda1* mutant.

### 3.2 Chitin deacetylation in the subperipheral zone

Chitosan was also found in the cell walls of vegetative hyphae located in the subperipheral zone of colonies. Here, chitosan localized not only to the septa and lateral walls of hyphae but occasionally also to hyphal tips, although not exclusively so. Chitin deacetylation in these cells did not require Cda1 but did require Cda4 (and perhaps to a lesser extent Cda5), two predicted secreted proteins with putative single transmembrane domains. Although the staining of chitosan in these hyphae was reduced in the Δ*cda4* and ΔΔ*cda4*/*cda5* strains, the loss of chitosan had little impact on radial growth or hyphal morphology. This suggests that chitin deacetylation is not required for cell wall integrity or morphogenesis in these hyphae. However, we cannot rule out compensatory changes in cell wall composition, which may have masked the effects of chitosan loss. The deletion strains did appear to be more sensitive to caffeine, but even here, the reduction in growth was marginal. Additionally, because caffeine has pleiotropic effects on cells (Levin, 2011), the increased sensitivity of the deletion strains to this chemical does not necessarily provide any particular insight into the role played by Cda4 and Cda5. However, numerous studies have found that mutations in components of the cell wall integrity signalling pathway or in genes with roles in cell wall biogenesis are also more sensitive to caffeine (Levin, 2011). The change in cell wall composition caused by deletion of *CDA4* and *CDA5* most likely causes the increased caffeine sensitivity, but the mechanism behind this is unknown.

Unlike Cda1:mCherry, the Cda4:mCherry fusion localized intracellularly, although the exact identity of the intracellular compartments is unknown. Nevertheless, this suggests that Cda4 may not deacetylate existing microfibrillar chitin *in situ* in the cell wall, although the presence of very low concentrations of Cda4 at the plasma membrane cannot be ruled out. Instead, it is possible that Cda4 may deacetylate nascent chitin chains in collaboration with a particular chitin synthase, that is, chitin is deacetylated as it is synthesized. Although Cda4:mCherry did not colocalize with Chs1:eGFP, it is hypothesized that Cda4 may colocalize with another chitin synthase (perhaps Chs2, Chs3, or Chs4) in intracellular vesicles (chitosomes). Cda4 may therefore be cotransported to the plasma membrane with a chitin synthase, a possibility that requires further investigation, especially in light of recent findings showing the cotransport of glucan synthases and chitin synthases in Ustilago maydis (Schuster et al*.*, 2016). The transmembrane domain of Cda4 presumably allows for close association between the CDA and a chitin synthase. Such membrane association has previously been found to be necessary for chitin deacetylation by Cda2 in *C. neoformans* (Gilbert, Baker, Specht, & Lodge, 2012).

### 3.3 Hydrolysis of chitosan in M. oryzae

Chitosan is clearly present in the cell wall of a number of distinct cell types in the *M. oryzae* life cycle, with apparently disparate roles. As part of this study, we also examined the role played by chitosanase, to gain insight into the relationship between chitin deacetylation and the hydrolysis of chitosan.

Unexpectedly, the expression of *CSN* did not coincide with the deacetylation of chitin over the life cycle of *M. oryzae*. Expression was highest during vegetative growth but was very low, or even absent, during appressorium development and *in planta* growth. This is despite the fact that significant concentrations of chitosan known to be present in the cell wall during these processes (Geoghegan & Gurr, 2016). The hydrolysis of chitosan may therefore presumably not always a requirement during development. Yet, although there is only a single chitosanase, this does not necessarily mean that there is only a single enzyme with chitosanase activity. Fungal chitosan is typically only 70%–90% deacetylated (Chatterjee & Guha, 2014; Munoz, Valencia, Valderruten, Ruiz‐Durantez, & Zuluaga, 2015; Pochanavanich & Suntornsuk, 2002; Yen & Mau, 2007), meaning that some GlcNAc residues remain in the polymer. Both chitosanases and chitinases are able to hydrolyze a mixed GlcN–GlcNAc linkage (Hartl et al*.*, 2012), and so chitinases are able to hydrolyze chitosan to some degree, depending on the degree of deacetylation. Thus, from the perspective of cell wall remodelling by cell wall hydrolases, it is difficult to make a clear distinction between chitin and chitosan. Any remodelling of chitosan required during cellular morphogenesis may just as well be performed by chitinases as by chitosanase. Such redundancy between chitinases and chitosanases could also explain a number of other observations, including the lack of chitosanases in some fungi and the normal growth of the *M. oryzae* Δ*csn* strain on chitosan. This could also help to explain the lack of any growth or morphogenic defects observed upon *CSN* deletion or knockdown in *M. oryzae*, *A. fumigatus* (Beck et al., 2014)*, F. solani* (Liu et al*.*, 2010), or *N. crassa* (Maddi, Dettman, Fu, Seiler, & Free, 2012). The presence of as yet uncharacterized enzymes with chitosanase activity is also a possibility.

There are also other possible reasons for the lack of chitosanase expression in certain developmental stages. For example, it may reflect differences in the mode of chitin deacetylation: We hypothesize that chitin deacetylation can occur either postsynthesis or coincident with chitin synthesis. There may therefore be different requirements for chitosan hydrolysis, dependent upon the mode of chitin deacetylation. Last, the inherent properties of chitosan as a hydrophilic, flexible polysaccharide could also mean that hydrolysis is not required to render it pliable in the same way as chitin, for example. Clearly, there are many questions that require further investigation in this area.

In summary, we have uncovered novel information regarding the roles of chitin deacetylation and chitosan hydrolysis in *M. oryzae*. First, chitosan was found to be a novel component of the cell wall in vegetative hyphae. Second, the fluorescent tagging of CDAs expressed during vegetative growth revealed two distinct modes of chitin deacetylation occurring in distinct zones of colonies. These may reflect different roles for chitosan depending on the developmental stage in question. Precisely what these roles may be remains unclear. Significantly, we found no evidence for a link between chitin deacetylation and morphogenesis during vegetative growth: Deletion or ectopic expression of CDAs did not result in detectable morphogenic defects, and localization of chitosan was not consistent with a morphogenic role. Similarly, there was no evidence for a role for chitosanase in cell wall remodelling during morphogenesis, and the role of this enzyme also remains unclear. Further work is therefore required to determine whether there is a relationship between chitin deacetylation, chitosan hydrolysis, cell wall flexibility, and morphogenesis. Nevertheless, the findings as they are represent a significant advance in our understanding of the biosynthesis and function of this important cell wall polysaccharide in fungi.

## 4 EXPERIMENTAL PROCEDURES

### 4.1 Fungal strains and growth conditions

The WT rice pathogenic *M. oryzae* strain Guy11 and mutant strains were cultured at 24 °C with a 14‐hr light 10‐hr dark cycle. Strain maintenance and composition of media were essentially as described by Talbot et al. (Talbot, Ebbole, & Hamer, 1993). For evaluating growth of strains on solid media under stress conditions, CM was supplemented with different treatments as follows: 40 μg/ml CFW, 100 μg/ml Congo Red, 5 mM H_2_O_2_, 1 M sorbitol, 2.5 mM caffeine, 2 g/L CuSO_4_, 0.005% (*w*/*v*) SDS, or 1 M NaCl. Radial growth was measured after a 10‐day incubation as described above.

### 4.2 Reverse transcription‐PCR

RNA was extracted from liquid cultures of the Guy11 strain growing in CM, using the Qiagen RNeasy RNA extraction kit, according to manufacturer's instructions. Reverse transcription and quantitative PCR analysis were performed exactly as described previously (Geoghegan & Gurr, 2016). Primers (listed in Table S1 (40–61) were designed to span an intron where possible (not all *CDA* genes have introns), and efficiency of the primers was 85%–104% (average efficiency 93.6%). No amplification was observed in samples that had not been reverse transcribed (−RT control) or in samples without RNA (no template control (NTC)). Relative transcript abundance was calculated by the efficiency correction method (REF) as follows: Abundance = E_Target_^(Ct(reference) − Ct(target))^.

### 4.3 Targeted deletion of M. oryzae CDAs

Single *CDA* deletion strains were generated by replacing the coding sequences of *CDA1* (*MGG_14966*), *CDA4* (*MGG_08774*), *CDA5* (*MGG_01868*), and *CSN* (*MGG_07656*) with a hygromycin resistance cassette (for *CDA1* and *CDA5*) or a bialaphos resistance cassette (for *CDA4* and *CSN*). Briefly, sequences flanking the target genes (~1.5 kb upstream and ~1.1 kb downstream) were amplified by PCR, using primers 5–8 for *CDA1*, 9–12 for *CDA4*, 13–16 for *CDA5*, and 17–20 for *CSN* (primers are listed in Table S1). These fragments were joined to the resistance gene by overlapping PCR, using primer pairs 5/4 and 3/8 for *CDA1*, 9/2 and 1/12 for *CDA4*, 13/4 and 3/16 for *CDA5*, and 17/2 and 1/20 for *CSN*. The final, complete construct was made by overlapping PCR, amplifying the products from the previous reactions using primer pairs 5/8 for *CDA1*, 9/12 for *CDA4*, and 13/16 for *CDA5*. The complete *CSN* deletion cassette could not be amplified with the requisite purity and so was used in two overlapping fragments (see Figure S8). The final PCR products were used directly for DNA‐mediated protoplast transformation of WT Guy11 strain following protocols described by Talbot et al. (Talbot et al*.*, 1993). Putative transformants were selected on minimal medium supplemented with 300 μg/ml hygromycin B (Calbiochem, Merck, Darmstadt, Germany) or defined complex medium supplemented with 60 μg/ml bialaphos (Goldbio, St. Louis, MO, USA). Deletion of the target gene was confirmed by both PCR and Southern blot analysis, as described in Samalova et al. (Samalova et al*.*, 2013).

Double *CDA* deletion strains were generated in the Δ*cda4* background strain. The coding sequence of *CDA5* was replaced with a hygromycin resistance cassette. The deletion construct was made as described above. The *CDA5* deletion cassette was transformed directly into protoplasts of the Δ*cda4* strain, to generate the ΔΔ*cda4*/*cda5* mutant. Putative transformants were selected on minimal medium supplemented with 300 μg/ml hygromycin B. Deletion strains were confirmed as above.

### 4.4 Cloning of fluorescently tagged CDAs, CSN, and promoter fusion

Standard molecular techniques (Ausubel et al*.*, 1999) were used to prepare the complementation constructs with fluorescently tagged *CDAs* and *CSN.* A set of transformation vectors based on pUCAP was generated as described in Samalova et al. (Samalova, Meyer, Gurr, & Fricker, 2014). The vectors contain polyadenylation signal pATrpC and either bialophos or hygromycin resistance marker that was cloned into recreated *Sal*I sites using primer pairs 1/2 or 3/4, respectively (see Table S1 and Figure S15). For PCR amplification of *CDA1*, *CDA4, CHS1* (*MGG_01868*), or *CSN*, primer pairs 21/22, 23/24, 27/28, or 25/26 were used, respectively. Genomic DNA from the WT strain Guy11 was used as a template and amplified using Herculase DNA polymerase (Agilent). This resulted in amplification of the coding sequence of the genes (without stop codons), together with up to 2 kb of native promoter sequence, depending on proximity of neighbouring genes. For the mCherry fusions, the PCR products were cloned into the *Asc*I sites of the vector described above (Figure S15), creating C‐terminal mCherry fusions. For eGFP fusions, mCherry was replaced with eGFP, and the PCR products cloned into the *Asc*I–*Sbf*I sites.

For ectopic expression of *CDA1*:mCherry, the promoter sequence of *EF1α* (*MGG_03641*) was amplified using primer pair 37/38 (Table S1). The coding sequence of *CDA1* was amplified using primer pair 39/22. The resulting DNA fragments were joined by overlapping PCR using primer pair 37/22, and the final construct cloned into the *Asc*I site of the vector (Figure S15).

### 4.5 Confocal imaging

For imaging of infection structures, conidia (2.5 × 10^5^ ml^−1^) of Guy11 and fluorescently tagged strains were collected from 10‐day‐old plates and inoculated in 50‐μl droplets onto hydrophobic glass coverslips or onion peels as described in Samalova et al. (Samalova et al*.*, 2014) and incubated for specified times in the growth chamber. For imaging of mycelial growth, conidia were incubated into liquid CM and incubated in the dark at 24 °C for ~72 hr, with shaking at 150 rpm. The resulting mycelial pellets could then be removed for imaging. Alternatively, a sterile glass coverslip was overlayed with CM and placed at the edge of a colony growing on solid medium. The fungus was allowed to grow over the coverslip for 24–72 hr, and then the coverslip could be removed for imaging.

For viewing mCherry fluorescence, the samples were viewed using the C‐Apochromat 40×/1.2 water corrected objective lens of a Zeiss LSM 510 Meta confocal microscope at 543‐nm excitation from the HeNe laser and emission collected with an LP585 filter. For dual imaging of OGA488 and mCherry or eGFP and mCherry, samples were viewed using a C‐Apochromat 40×/1.2 water corrected objective lens of a Zeiss LSM 510 Meta confocal microscope. Dual excitation at 488 nm and 543 nm was provided by Argon and Helium–Neon lasers, respectively. Emitted light was collected with BP500 to 530‐ and LP585‐nm filters.

CFW staining was performed as follows: Mycelial pellets were washed briefly with dH_2_O, then incubated on ice with 0.05% CFW for 20 min. Samples were washed 2–3 times with dH_2_O and viewed using the Zeiss LSM510 microscope as above, with 405‐nm excitation and emission collected with an LP420 filter.

OGA488 staining was performed essentially as described in Geoghegan and Gurr (2016). Samples (mycelial pellets and coverslips that were overgrown with hyphae or had previously been inoculated with conidia) were washed briefly with 25 mM MES (pH 5.6) and incubated with OGA488, a generous gift from William Willats (Mravec et al*.*, 2014; diluted 1/1000 in 25 mM MES) for 15 min on ice. This was followed by 2–3 brief washes with 25 mM MES, after which the samples were viewed using the C‐Apochromat 40×/1.2 water corrected objective lens of a Zeiss LSM 510 Meta confocal microscope, at 488‐nm excitation and emission collected with an LP505 filter.

Staining of hyphae with FITC‐labelled wheat germ agglutinin (Sigma‐Aldrich, UK) was performed as follows: Mycelial pellets of the WT or Δ*cda1* strains were incubated with 2% bovine serum albumin in phosphate‐buffered saline (PBS) on ice, then washed 3 times with PBS/T (PBS + 0.05% Tween 20). Pellets were then incubated with 10 μg/ml ConA‐FITC for 3 hr on ice, then washed 3 times with PBS/T. Samples were viewed using the C‐Apochromat 40×/1.2 water corrected objective lens of a Zeiss LSM 510 Meta confocal microscope, at 488‐nm excitation and emission collected with an LP505 filter.

Staining with the monoclonal anti‐chitosan antibody mAbG7, a generous gift from Stefan Schillberg (Schubert et al*.*, 2010), was performed as described previously (Geoghegan & Gurr, 2016).

Eosin Y staining was performed as described by Baker et al*.* (2007). Briefly, conidia were harvested, pelleted by centrifugation at 2,850 g in a Beckman Coulter Allegra X‐15R, and then resuspended in McIlvaine buffer (pH 6). Conidial concentration was adjusted to 1 × 10^6^ conidia per milliliter. To 500 μl of conidial suspension, 10‐μl eosin Y (5 mg/ml) was added and incubated on ice for 30 min. Conidia were washed by centrifugation of the labelled spore suspension at 16,000 g for 5 min, removal of the supernatant, and resuspension in 1‐ml McIlvaine buffer. This was repeated 3 times, except after the last wash, conidia were resuspended in 100 ml of McIlvaine buffer. Labelled conidia could then be viewed with an Olympus BX50 microscope, using a NIBA filter.

### 4.6 Pathogenicity and infection‐related morphogenesis assays

Conidial germination and appressorium development were assessed at 1, 8, 16, or 24 hpi by following germling differentiation on hydrophobic glass coverslips (Gerhard Menzel, Glasbearbeitungswerk GmbH & Co., Braunschweig, Germany). Conidia (2.5 × 10^5^ ml^−1^) of the Guy11 and mutant strains were inoculated in 50‐μl droplets onto hydrophobic glass coverslips and incubated in the growth chamber for the specified time. Samples were viewed under an Olympus BX50 microscope, and ~500 germlings in three independent experiments counted per strain/time point.

Leaf infection assays were performed on blast‐susceptible, 21‐day‐old seedlings of rice (Oryza sativa L.) cultivar CO39. Assays on detached leaves were performed as described in Samalova et al*.* (2013). For assays on whole plants, 21‐day‐old seedlings of rice (O. sativa L.) cultivar CO39 were spray inoculated with 4 ml of conidial suspension at three different concentrations (1.25 × 10^5^, 6.25 × 10^4^, or 3.13 × 10^4^ conidia per milliliter, in 0.2% [*w*/*v*] gelatine water). A mock inoculation of 0.2% (*w*/*v*) gelatine water was included as a negative control. Infection was assessed 4 days later.

## Supporting information


**Data S1.** **Figure S1. Antibody staining of chitosan in vegetative hyphae. A & B**) Mycelial pellets of *M.oryzae* stained with the monoclonal anti‐chitosan antibody mAbG7. **C**) Secondary antibody only control, showing lack of staining. Scale bars: 20 μm.
**Figure S2. Domain architecture of CDA1, CDA4 and CDA5.** CDA = Chitin deacetylase, CBD = Chitin binding domain.
**Figure S3. PCR analysis of *CDA* deletion strains. A**) Schematic of targeted deletion strategy. Homologous recombination replaces the target gene with a gene imparting antibiotic resistance. **B**) PCR analysis of deletion strains. Putative deletion strains were screened by PCR to confirm the absence of the target gene (P1), and the integration of the deletion construct at the desired locus (P2 & P3). Position of primers shown in A.
**Figure S4. Southern Blot analysis of *CDA* deletion strains.** Blots containing restriction digested gDNA of putative deletion strains were hybridised with α‐^32^P labelled DNA homologous to the hygromycin (*HYG*) (for *CDA1* and *CDA5*) or bialaphos (*BAR*) (for *CDA4*) resistance genes. The cartoon above each blot shows the expected band size based upon the positions of the restriction enzymes sites at each locus. Size markers show band size in kilobases (kb). Successful single insertions were obtained for each of the 3 genes. In the ΔΔ*cda4*/*cda5* strain, cross‐hybridisation (band at ~20 kb) is observed between the *HYG* probe and the *BAR* gene used in the Δ*cda4* background strain. This is due to a common promoter sequence used in both the *BAR* and *HYG* resistance cassettes.
**Figure S5. Radial growth of Δ*cda1* strain under different stress conditions, and pathogenic development. A**) Table of colony diameters (mm) (± SD, *n* = 3) of the WT and ∆*cda1* strains grown on a range of different solid media, after 10 days incubation. **B**) Representative pictures of the Δ*cda1* strain growing on solid medium, taken after 10 days incubation. CM = Complete medium, MM = minimal medium, CFW = Calcofluor White, CR = Congo Red, SDS = Sodium Dodecyl sulphate, MM‐N = minimal medium without nitrogen, MM – C = minimal media without carbon. **C**) Appressoria of WT and Δ*cda1* at 24 hpi on an artificial inductive surface. The deletion strain shows normal appressorium development. Scale bars: 20 μm. **D**) Pathogenicity of Δ*cda1* on rice leaves. Leaves inoculated with conidia of Δ*cda1* showed similar lesion density to the WT strain. A mock inoculation of 0.2% gelatine (*w*/*v*) was included as a negative control. Scale bars: 0.5 cm.
**Figure S6. N‐terminally mCherry tagged Cda4 localizes intracellularly in vegetative hyphae. A**) Confocal microscopy images showing weak fluorescence of strains expressing mCherry:Cda4. The mCherry was cloned into position after the signal peptide of Cda4, thereby creating an N‐terminal fusion in the mature Cda4 protein. **B**) Hyphae of the WT strain, showing lack of fluorescence. Scale bars: 20 μm.
**Figure S7. mCherry tagged Cda4 does not colocalize with eGFP tagged chitin synthase I.** Chs1:eGFP localized to the cell periphery at hyphal tips, and also to intracellular punctae (possible `chitosomes'). Some colocalization with Cda4:mCherry was observed in larger intracellular bodies, but not at the cell periphery. Scale bars: 20 μm.
**Figure S8. Radial growth of Δ*cda4,* Δ*cda5* and ΔΔ*cda4*/*cda5* strains under different conditions. A**) Table of colony diameters (mm) (± SD, *n* = 3) of WT and deletion strains on solid media, after 10 days incubation. Two independent ΔΔ*cda4*/*cda5* were tested (numbered 13 and 23). * = statistically significant difference, according to a Student's T‐test (*p* < 0.05). **B**) Representative images of the Δ*cda4,* Δ*cda5* and ΔΔ*cda4*/*cda5* strains growing on solid media, taken after 10 days incubation. Two independent ΔΔ*cda4*/*cda5* lines are shown. CM = Complete medium, MM = minimal medium, CFW = Calcofluor White, CR = Congo Red, SDS = Sodium Dodecyl sulphate, MM‐N = minimal medium without nitrogen, MM‐C = minimal media without carbon.
**Figure S9: Pathogenic development in the Δ*cda4*, Δ*cda5* and ΔΔ*cda4*/*cda5* strains**. **A**) Appressoria of the WT and deletion strains at 24 hpi on a hydrophobic glass surface, showing normal development in all strains. Scale bars: 20 μm. **B**) Pathogenicity of the deletion strains on rice leaves. Leaves inoculated with conidia of either the WT or deletion strains showed similar lesion densities. A mock inoculation of 0.2% gelatine (*w*/*v*) was included as a negative control. Scale bars: 5 mm.
**Figure S10. Sequence information of the *M.oryzae* chitosanase. A**) Protein domain architecture of chitosanase. Green box = Signal peptide. **B**) Protein sequence of the *M.oryzae* chitosanase. The signal peptide, chitosanase domain and repeat sequences have been annotated and color‐coded to correspond with the diagram in **A. C**) Alignment of fungal chitosanase sequences, showing conservation of the key catalytic aspartate and glutamate residues (green boxes). **D**) Prediction of protein disorder using PONDR VSL2 (purple line) and VL‐XT (red line).
**Figure S11. Localization of the *eGFP* fusion of *CSN*.** Fluorescence of the Csn:eGFP fusion, showing apparent cytoplasmic localization of the fusion protein in vegetative hyphae. Scale bar: 50 μm.
**Figure S12. PCR and Southern blot analysis of Δ*csn***. **A**) Schematic of targeted deletion strategy. A deletion cassette in two overlapping parts was used to replace the target gene (*CSN*) with one imparting antibiotic resistance (*BAR*). **B**) PCR analysis of putative deletion strains. Position of primers used is shown in A. **C**) Southern blot analysis of putative deletion strains. Restriction digested (*Pst*I) gDNA of putative deletion strains was hybridised with α‐^32^P labelled DNA homologous to *BAR*. A single band of 3.8 kb was expected in successful deletion strains. Size markers show band size in kilobases (Kb). Four of the transformants were successful deletion strains (numbered 9, 24, 46). Of these, 9 and 46 were chosen at random for characterization.
**Figure S13. Chitosan staining and autolysis in Δ*csn.* A**) Labelling of chitosan on vegetative hyphae grown on solid medium, using the chitosan‐specific probe OGA488. Scale bars: 50 μm. **B**) Staining of germlings with anti‐chitosan antibody mAbG7. Germlings had been germinated on an artificial inductive surface for 16 hr prior to labelling. Both germling morphology and chitosan labelling were unaffected in the Δ*csn* strain. Scale bars: 20 μm. **C**) Eosin Y staining of conidia, which was unaffected in Δ*csn*. Scale bars: 20 μm. **D**) Growth of the Δ*csn* mutant (grey bars) in liquid medium with 0.33% chitosan (*w*/*v*) as a sole carbon source, showing similar accumulation of biomass to the WT strain (white bars). Error bars show SD, *n* = 3. **E**) The Δ*csn* mutant was grown in liquid minimal medium for 21 days, with biomass measurements taken every 4 days. Growth of the Δ*csn* mutant (grey lines, two independent transformant lines shown) was similar to the WT (black line). Error bars show SD, *n* = 2.
**Figure S14. Radial growth of the Δ*csn* strain under different stress conditions, and pathogenic development. A**) Table of colony diameters (mm) (± SD, *n* = 3) of WT and ∆*csn* strains on solid media, after 10 days incubation. Two independent deletion strains were tested, numbered 9 and 46. * = statistically significant difference, according to a Student's T‐test (*p* < 0.05). **B**) Pictures of the Δ*csn* strain growing on solid medium, taken after 10 days incubation. Two independent Δ*csn* lines are shown (named 9 and 46). CM = Complete medium, MM = minimal medium, CFW = Calcofluor White, CR = Congo Red, SDS = Sodium Dodecyl sulphate, MM‐N = minimal medium without nitrogen, MM‐C = minimal media without carbon, NaCl = sodium chloride. **C**) Table showing conidial germination (at 1 hpi) and appressorium development (at 8 hpi) on an artificial inductive surface, showing no differences between WT and Δ*csn*. Figures are ±SD, *n* = 3. **D**) Pathogenicity of the csn strain on barley leaves, showing similar lesion numbers to the WT strain (**E**). A mock inoculation of 0.2% gelatine was included as a negative control. Error bars show SD, *n* = 3.
**Figure S15. Map of plasmid used for cloning of fluorescently tagged proteins in this study.**
**Table S1. Primers used in this study.**
**Table S2. Occurrence and distribution of chitosanases in fungi.** Table showing the number of putative chitosanase sequences present in genomes of 75 fungal species. Chitosanases are absent in the Basidiomycota, Chytridiomycota (not shown) and Zygomycota (not shown).
