# Supplementary material for: Investigating chitin deacetylation and chitosan hydrolysis during vegetative growth in Magnaporthe oryzae
Source: Cell Microbiol. 2017 Apr 26;19(9):e12743. doi: 10.1111/cmi.12743 (PMC5573952; doi:10.1111/cmi.12743)
Supplement: Supplementary file 1 — Data S1. Figure S1. Antibody staining of chitosan in vegetative hyphae. A & B) Mycelial pellets of M.oryzae stained with the monoclonal anti‐chitosan antibody mAbG7. C) Secondary antibody only control, showing lack of staining. Scale bars: 20 μm. Figure S2. Domain architecture of CDA1, CDA4 and CDA5. CDA = Chitin deacetylase, CBD = Chitin binding domain. Figure S3. PCR analysis of CDA deletion strains. A) Schematic of targeted deletion strategy. Homologous recombination replaces the target gene with a gene imparting antibiotic resistance. B) PCR analysis of deletion strains. Putative deletion strains were screened by PCR to confirm the absence of the target gene (P1), and the integration of the deletion construct at the desired locus (P2 & P3). Position of primers shown in A. Figure S4. Southern Blot analysis of CDA deletion strains. Blots containing restriction digested gDNA of putative deletion strains were hybridised with α‐32P labelled DNA homologous to the hygromycin (HYG) (for CDA1 and CDA5) or bialaphos (BAR) (for CDA4) resistance genes. The cartoon above each blot shows the expected band size based upon the positions of the restriction enzymes sites at each locus. Size markers show band size in kilobases (kb). Successful single insertions were obtained for each of the 3 genes. In the ΔΔcda4/cda5 strain, cross‐hybridisation (band at ~20 kb) is observed between the HYG probe and the BAR gene used in the Δcda4 background strain. This is due to a common promoter sequence used in both the BAR and HYG resistance cassettes. Figure S5. Radial growth of Δcda1 strain under different stress conditions, and pathogenic development. A) Table of colony diameters (mm) (± SD, n = 3) of the WT and ∆cda1 strains grown on a range of different solid media, after 10 days incubation. B) Representative pictures of the Δcda1 strain growing on solid medium, taken after 10 days incubation. CM = Complete medium, MM = minimal medium, CFW = Calcofluor White, CR = Congo Red, SDS = Sodium [file CMI-19-na-s001.zip › Supplementary figure captions.docx]

Supplementary figure captions

**Figure S1. Antibody staining of chitosan in vegetative hyphae. A & B**) Mycelial pellets of *M.oryzae* stained with the monoclonal anti-chitosan antibody mAbG7. **C**) Secondary antibody only control, showing lack of staining. Scale bars: 20 µm.

**Figure S2. Domain architecture of CDA1, CDA4 and CDA5.** CDA = Chitin deacetylase, CBD = Chitin binding domain.

**Figure S3. PCR analysis of *CDA* deletion strains. A**) Schematic of targeted deletion strategy. Homologous recombination replaces the target gene with a gene imparting antibiotic resistance. **B**) PCR analysis of deletion strains. Putative deletion strains were screened by PCR to confirm the absence of the target gene (P1), and the integration of the deletion construct at the desired locus (P2 & P3). Position of primers shown in A.

**Figure S4. Southern Blot analysis of *CDA* deletion strains.** Blots containing restriction digested gDNA of putative deletion strains were hybridised with α-^32^P labelled DNA homologous to the hygromycin (*HYG*) (for *CDA1* and *CDA5*) or bialaphos (*BAR*) (for *CDA4*) resistance genes. The cartoon above each blot shows the expected band size based upon the positions of the restriction enzymes sites at each locus. Size markers show band size in kilobases (kb). Successful single insertions were obtained for each of the 3 genes. In the ΔΔ*cda4/cda5* strain, cross-hybridisation (band at ~20kb) is observed between the *HYG* probe and the *BAR* gene used in the Δ*cda4* background strain. This is due to a common promoter sequence used in both the *BAR* and *HYG* resistance cassettes.

**Figure S5. Radial growth of Δ*cda1* strain under different stress conditions, and pathogenic development. A**) Table of colony diameters (mm) (± SD, n = 3) of the WT and ∆*cda1* strains grown on a range of different solid media, after 10 days incubation. **B**) Representative pictures of the Δ*cda1* strain growing on solid medium, taken after 10 days incubation. CM = Complete medium, MM = minimal medium, CFW = Calcofluor White, CR = Congo Red, SDS = Sodium Dodecyl sulphate, MM-N = minimal medium without nitrogen, MM – C = minimal media without carbon. **C**) Appressoria of WT and Δ*cda1* at 24 hpi on an artificial inductive surface. The deletion strain shows normal appressorium development. Scale bars: 20 µm. **D**) Pathogenicity of Δ*cda1* on rice leaves. Leaves inoculated with conidia of Δ*cda1* showed similar lesion density to the WT strain. A mock inoculation of 0.2% gelatine (w/v) was included as a negative control. Scale bars: 0.5 cm.

**Figure S6. N-terminally mCherry tagged Cda4 localizes intracellularly in vegetative hyphae. A**) Confocal microscopy images showing weak fluorescence of strains expressing mCherry:Cda4. The mCherry was cloned into position after the signal peptide of Cda4, thereby creating an N-terminal fusion in the mature Cda4 protein. **B**) Hyphae of the WT strain, showing lack of fluorescence. Scale bars: 20 µm.

**Figure S7. mCherry tagged Cda4 does not colocalize with eGFP tagged chitin synthase I.** Chs1:eGFP localized to the cell periphery at hyphal tips, and also to intracellular

punctae (possible `chitosomes'). Some colocalization with Cda4:mCherry was observed in larger intracellular bodies, but not at the cell periphery. Scale bars: 20 µm.

**Figure S8. Radial growth of Δ*cda4,*  Δ*cda5* and ΔΔ*cda4/cda5* strains under different conditions. A**) Table of colony diameters (mm) (± SD, n = 3) of WT and deletion strains on solid media, after 10 days incubation. Two independent ΔΔ*cda4/cda5* were tested (numbered 13 and 23). * = statistically significant difference, according to a Student’s T-test (p < 0.05). **B**) Representative images of the Δ*cda4,*  Δ*cda5* and ΔΔ*cda4/cda5* strains growing on solid media, taken after 10 days incubation. Two independent ΔΔ*cda4/cda5* lines are shown. CM = Complete medium, MM = minimal medium, CFW = Calcofluor White, CR = Congo Red, SDS = Sodium Dodecyl sulphate, MM-N = minimal medium without nitrogen, MM-C = minimal media without carbon.

**Figure S9: Pathogenic development in the Δ*cda4*, Δ*cda5* and ΔΔ*cda4/cda5***

**strains**. **A**) Appressoria of the WT and deletion strains at 24 hpi on a hydrophobic glass surface, showing normal development in all strains. Scale bars: 20 µm. **B**) Pathogenicity of the deletion strains on rice leaves. Leaves inoculated with conidia of either the WT or deletion strains showed similar lesion densities. A mock inoculation of 0.2% gelatine (w/v) was included as a negative control. Scale bars: 5 mm.

**Figure S10. Sequence information of the *M.oryzae* chitosanase. A**) Protein domain architecture of chitosanase. Green box = Signal peptide. **B**) Protein sequence of the *M.oryzae* chitosanase. The signal peptide, chitosanase domain and repeat sequences have been annotated and color-coded to correspond with the diagram in **A.** **C**) Alignment of fungal chitosanase sequences, showing conservation of the key catalytic aspartate and glutamate residues (green boxes). **D**) Prediction of protein disorder using PONDR VSL2 (purple line) and VL-XT (red line).

**Figure S11. Localization of the *eGFP* fusion of *CSN*.** Fluorescence of the Csn:eGFP fusion, showing apparent cytoplasmic localization of the fusion protein in vegetative hyphae. Scale bar: 50 µm.

**Figure S12. PCR and Southern blot analysis of Δ*csn***. **A**) Schematic of targeted deletion strategy. A deletion cassette in two overlapping parts was used to replace the target gene (*CSN*) with one imparting antibiotic resistance (*BAR*). **B**) PCR analysis of putative deletion strains. Position of primers used is shown in A. **C**) Southern blot analysis of putative deletion strains. Restriction digested (*Pst*I) gDNA of putative deletion strains was hybridised with α-^32^P labelled DNA homologous to *BAR*. A single band of 3.8kb was expected in successful deletion strains. Size markers show band size in kilobases (Kb). Four of the transformants were successful deletion strains (numbered 9, 24, 46). Of these, 9 and 46 were chosen at random for characterization.

**Figure S13. Chitosan staining and autolysis in Δ*csn.* A**) Labelling of chitosan on

vegetative hyphae grown on solid medium, using the chitosan-specific probe OGA488. Scale bars: 50µm. **B**) Staining of germlings with anti-chitosan antibody mAbG7. Germlings had been germinated on an artificial inductive surface for 16 hr prior to labelling. Both germling morphology and chitosan labelling were unaffected in the Δ*csn* strain. Scale bars: 20 µm. **C**) Eosin Y staining of conidia, which was unaffected in Δ*csn*. Scale bars: 20 µm. **D**) Growth of the Δ*csn* mutant (grey bars) in liquid medium with 0.33% chitosan (w/v) as a sole carbon source, showing similar accumulation of biomass to the WT strain (white bars). Error bars show SD, n=3. **E**) The Δ*csn* mutant was grown in liquid minimal medium for 21 days, with biomass measurements taken every 4 days. Growth of the Δ*csn* mutant (grey lines, two independent transformant lines shown) was similar to the WT (black line). Error bars show SD, n = 2.

**Figure S14. Radial growth of the Δ*csn* strain under different stress conditions, and pathogenic development. A**) Table of colony diameters (mm) (± SD, n = 3) of WT and ∆*csn* strains on solid media, after 10 days incubation. Two independent deletion strains were tested, numbered 9 and 46. * = statistically significant difference, according to a Student’s T-test (p < 0.05). **B**) Pictures of the Δ*csn* strain growing on solid medium, taken after 10 days incubation. Two independent Δ*csn* lines are shown (named 9 and 46). CM = Complete medium, MM = minimal medium, CFW = Calcofluor White, CR = Congo Red, SDS = Sodium Dodecyl sulphate, MM-N = minimal medium without nitrogen, MM-C = minimal media without carbon, NaCl = sodium chloride. **C**) Table showing conidial germination (at 1 hpi) and appressorium development (at 8 hpi) on an artificial inductive surface, showing no differences between WT and Δ*csn*. Figures are ±SD, n=3. **D**) Pathogenicity of the csn strain on barley leaves, showing similar lesion numbers to the WT strain (**E**). A mock inoculation of 0.2% gelatine was included as a negative control. Error bars show SD, n=3.

**Figure S15. Map of plasmid used for cloning of fluorescently tagged proteins in this study.**

**Table S1. Primers used in this study.**

**Table S2. Occurrence and distribution of chitosanases in fungi.** Table showing the number of putative chitosanase sequences present in genomes of 75 fungal species. Chitosanases are absent in the Basidiomycota, Chytridiomycota (not shown) and Zygomycota (not shown).
