# Supplementary figures and images for: Investigating chitin deacetylation and chitosan hydrolysis during vegetative growth in Magnaporthe oryzae
Source: Cell Microbiol. 2017 Apr 26;19(9):e12743. doi: 10.1111/cmi.12743 (PMC5573952; doi:10.1111/cmi.12743)

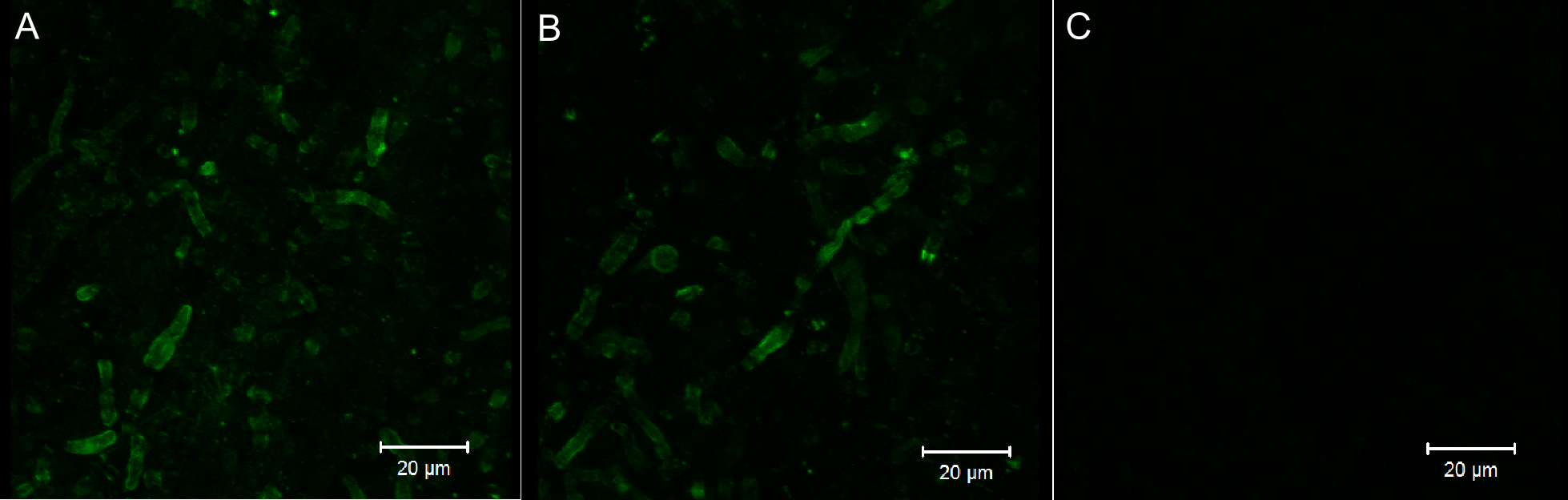

Supplement: Supplementary file 1 — Data S1. Figure S1. Antibody staining of chitosan in vegetative hyphae. A & B) Mycelial pellets of M.oryzae stained with the monoclonal anti‐chitosan antibody mAbG7. C) Secondary antibody only control, showing lack of staining. Scale bars: 20 μm. Figure S2. Domain architecture of CDA1, CDA4 and CDA5. CDA = Chitin deacetylase, CBD = Chitin binding domain. Figure S3. PCR analysis of CDA deletion strains. A) Schematic of targeted deletion strategy. Homologous recombination replaces the target gene with a gene imparting antibiotic resistance. B) PCR analysis of deletion strains. Putative deletion strains were screened by PCR to confirm the absence of the target gene (P1), and the integration of the deletion construct at the desired locus (P2 & P3). Position of primers shown in A. Figure S4. Southern Blot analysis of CDA deletion strains. Blots containing restriction digested gDNA of putative deletion strains were hybridised with α‐32P labelled DNA homologous to the hygromycin (HYG) (for CDA1 and CDA5) or bialaphos (BAR) (for CDA4) resistance genes. The cartoon above each blot shows the expected band size based upon the positions of the restriction enzymes sites at each locus. Size markers show band size in kilobases (kb). Successful single insertions were obtained for each of the 3 genes. In the ΔΔcda4/cda5 strain, cross‐hybridisation (band at ~20 kb) is observed between the HYG probe and the BAR gene used in the Δcda4 background strain. This is due to a common promoter sequence used in both the BAR and HYG resistance cassettes. Figure S5. Radial growth of Δcda1 strain under different stress conditions, and pathogenic development. A) Table of colony diameters (mm) (± SD, n = 3) of the WT and ∆cda1 strains grown on a range of different solid media, after 10 days incubation. B) Representative pictures of the Δcda1 strain growing on solid medium, taken after 10 days incubation. CM = Complete medium, MM = minimal medium, CFW = Calcofluor White, CR = Congo Red, SDS = Sodium [file CMI-19-na-s001.zip › FigureS1.tif]

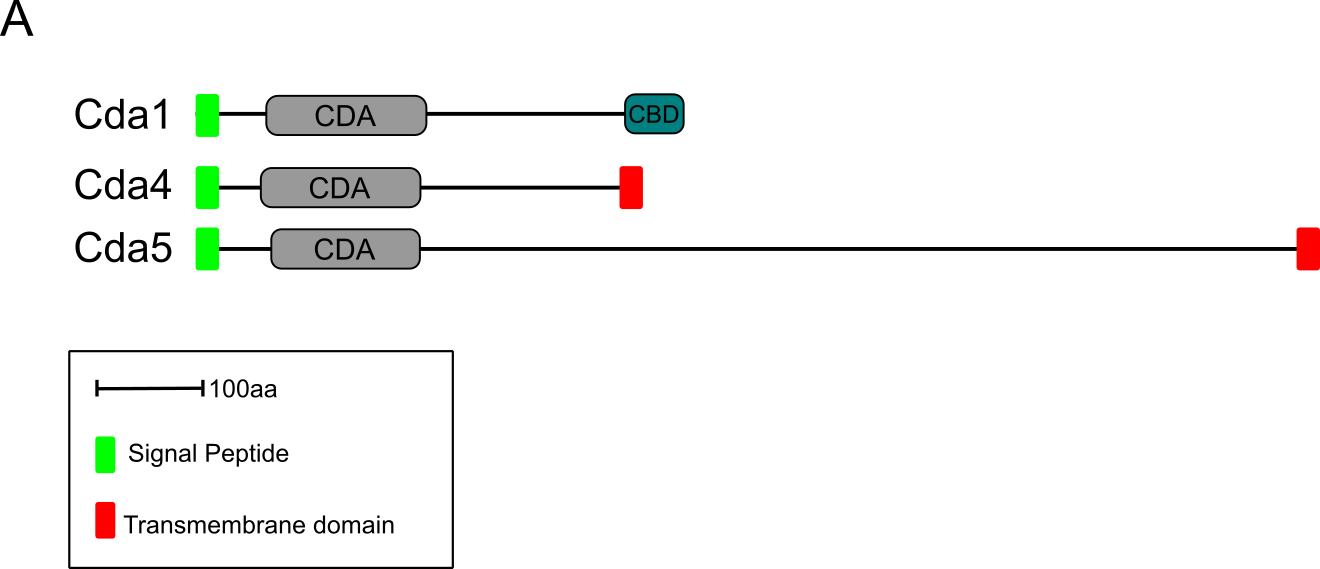

Supplement: Supplementary file 1 — Data S1. Figure S1. Antibody staining of chitosan in vegetative hyphae. A & B) Mycelial pellets of M.oryzae stained with the monoclonal anti‐chitosan antibody mAbG7. C) Secondary antibody only control, showing lack of staining. Scale bars: 20 μm. Figure S2. Domain architecture of CDA1, CDA4 and CDA5. CDA = Chitin deacetylase, CBD = Chitin binding domain. Figure S3. PCR analysis of CDA deletion strains. A) Schematic of targeted deletion strategy. Homologous recombination replaces the target gene with a gene imparting antibiotic resistance. B) PCR analysis of deletion strains. Putative deletion strains were screened by PCR to confirm the absence of the target gene (P1), and the integration of the deletion construct at the desired locus (P2 & P3). Position of primers shown in A. Figure S4. Southern Blot analysis of CDA deletion strains. Blots containing restriction digested gDNA of putative deletion strains were hybridised with α‐32P labelled DNA homologous to the hygromycin (HYG) (for CDA1 and CDA5) or bialaphos (BAR) (for CDA4) resistance genes. The cartoon above each blot shows the expected band size based upon the positions of the restriction enzymes sites at each locus. Size markers show band size in kilobases (kb). Successful single insertions were obtained for each of the 3 genes. In the ΔΔcda4/cda5 strain, cross‐hybridisation (band at ~20 kb) is observed between the HYG probe and the BAR gene used in the Δcda4 background strain. This is due to a common promoter sequence used in both the BAR and HYG resistance cassettes. Figure S5. Radial growth of Δcda1 strain under different stress conditions, and pathogenic development. A) Table of colony diameters (mm) (± SD, n = 3) of the WT and ∆cda1 strains grown on a range of different solid media, after 10 days incubation. B) Representative pictures of the Δcda1 strain growing on solid medium, taken after 10 days incubation. CM = Complete medium, MM = minimal medium, CFW = Calcofluor White, CR = Congo Red, SDS = Sodium [file CMI-19-na-s001.zip › FigureS2.tif]

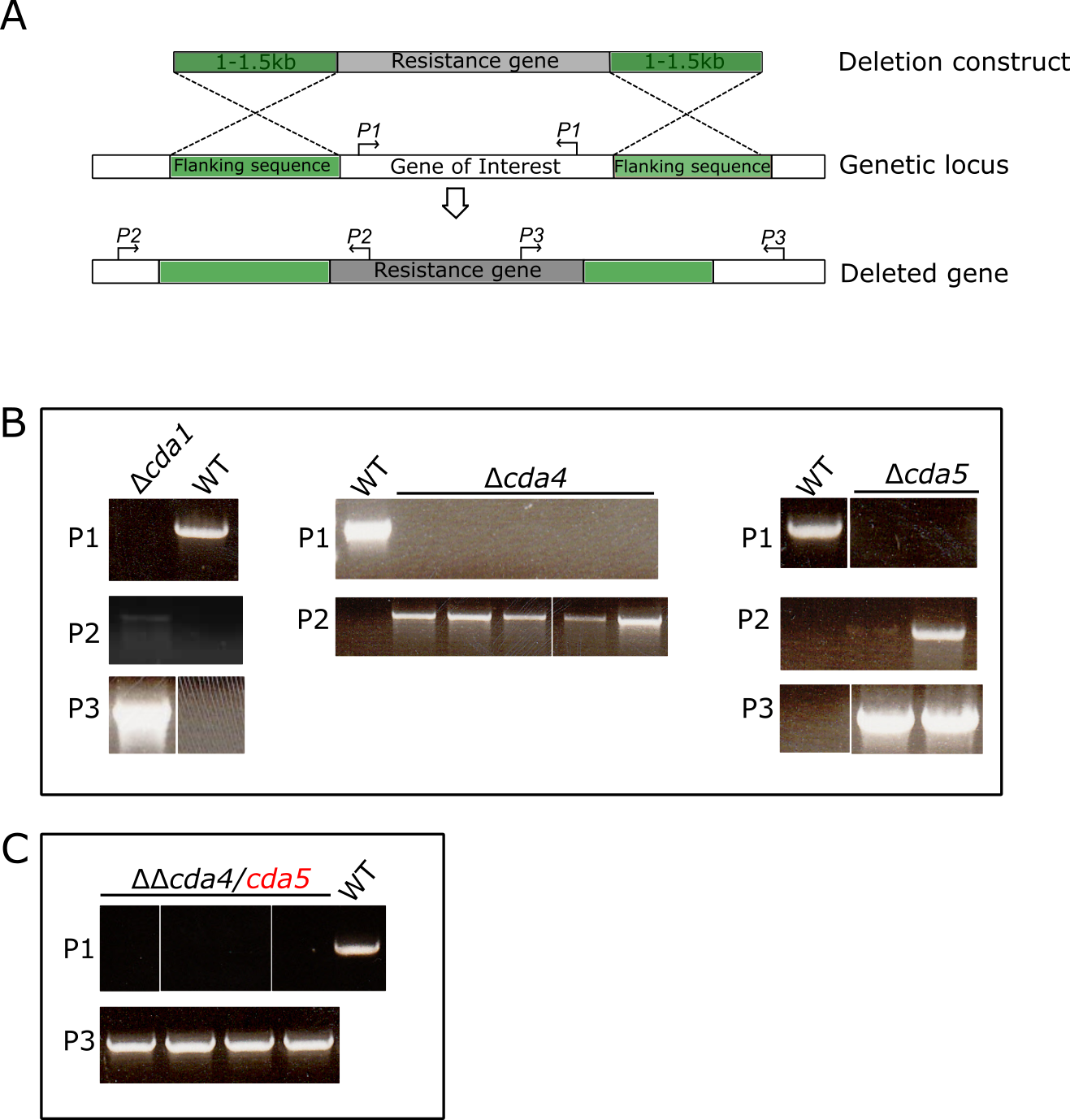

Supplement: Supplementary file 1 — Data S1. Figure S1. Antibody staining of chitosan in vegetative hyphae. A & B) Mycelial pellets of M.oryzae stained with the monoclonal anti‐chitosan antibody mAbG7. C) Secondary antibody only control, showing lack of staining. Scale bars: 20 μm. Figure S2. Domain architecture of CDA1, CDA4 and CDA5. CDA = Chitin deacetylase, CBD = Chitin binding domain. Figure S3. PCR analysis of CDA deletion strains. A) Schematic of targeted deletion strategy. Homologous recombination replaces the target gene with a gene imparting antibiotic resistance. B) PCR analysis of deletion strains. Putative deletion strains were screened by PCR to confirm the absence of the target gene (P1), and the integration of the deletion construct at the desired locus (P2 & P3). Position of primers shown in A. Figure S4. Southern Blot analysis of CDA deletion strains. Blots containing restriction digested gDNA of putative deletion strains were hybridised with α‐32P labelled DNA homologous to the hygromycin (HYG) (for CDA1 and CDA5) or bialaphos (BAR) (for CDA4) resistance genes. The cartoon above each blot shows the expected band size based upon the positions of the restriction enzymes sites at each locus. Size markers show band size in kilobases (kb). Successful single insertions were obtained for each of the 3 genes. In the ΔΔcda4/cda5 strain, cross‐hybridisation (band at ~20 kb) is observed between the HYG probe and the BAR gene used in the Δcda4 background strain. This is due to a common promoter sequence used in both the BAR and HYG resistance cassettes. Figure S5. Radial growth of Δcda1 strain under different stress conditions, and pathogenic development. A) Table of colony diameters (mm) (± SD, n = 3) of the WT and ∆cda1 strains grown on a range of different solid media, after 10 days incubation. B) Representative pictures of the Δcda1 strain growing on solid medium, taken after 10 days incubation. CM = Complete medium, MM = minimal medium, CFW = Calcofluor White, CR = Congo Red, SDS = Sodium [file CMI-19-na-s001.zip › FigureS3.tif]

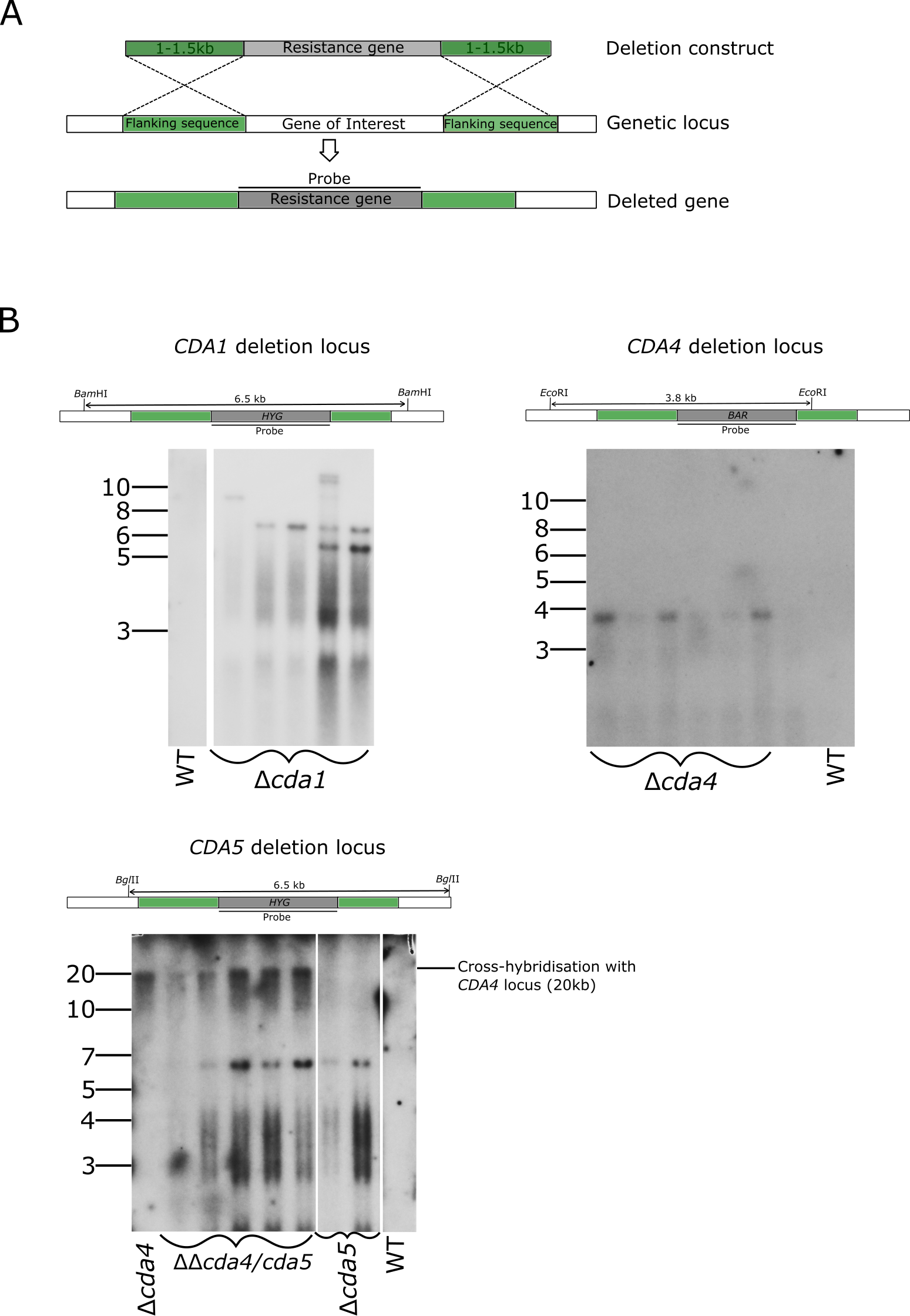

Supplement: Supplementary file 1 — Data S1. Figure S1. Antibody staining of chitosan in vegetative hyphae. A & B) Mycelial pellets of M.oryzae stained with the monoclonal anti‐chitosan antibody mAbG7. C) Secondary antibody only control, showing lack of staining. Scale bars: 20 μm. Figure S2. Domain architecture of CDA1, CDA4 and CDA5. CDA = Chitin deacetylase, CBD = Chitin binding domain. Figure S3. PCR analysis of CDA deletion strains. A) Schematic of targeted deletion strategy. Homologous recombination replaces the target gene with a gene imparting antibiotic resistance. B) PCR analysis of deletion strains. Putative deletion strains were screened by PCR to confirm the absence of the target gene (P1), and the integration of the deletion construct at the desired locus (P2 & P3). Position of primers shown in A. Figure S4. Southern Blot analysis of CDA deletion strains. Blots containing restriction digested gDNA of putative deletion strains were hybridised with α‐32P labelled DNA homologous to the hygromycin (HYG) (for CDA1 and CDA5) or bialaphos (BAR) (for CDA4) resistance genes. The cartoon above each blot shows the expected band size based upon the positions of the restriction enzymes sites at each locus. Size markers show band size in kilobases (kb). Successful single insertions were obtained for each of the 3 genes. In the ΔΔcda4/cda5 strain, cross‐hybridisation (band at ~20 kb) is observed between the HYG probe and the BAR gene used in the Δcda4 background strain. This is due to a common promoter sequence used in both the BAR and HYG resistance cassettes. Figure S5. Radial growth of Δcda1 strain under different stress conditions, and pathogenic development. A) Table of colony diameters (mm) (± SD, n = 3) of the WT and ∆cda1 strains grown on a range of different solid media, after 10 days incubation. B) Representative pictures of the Δcda1 strain growing on solid medium, taken after 10 days incubation. CM = Complete medium, MM = minimal medium, CFW = Calcofluor White, CR = Congo Red, SDS = Sodium [file CMI-19-na-s001.zip › FigureS4.tif]

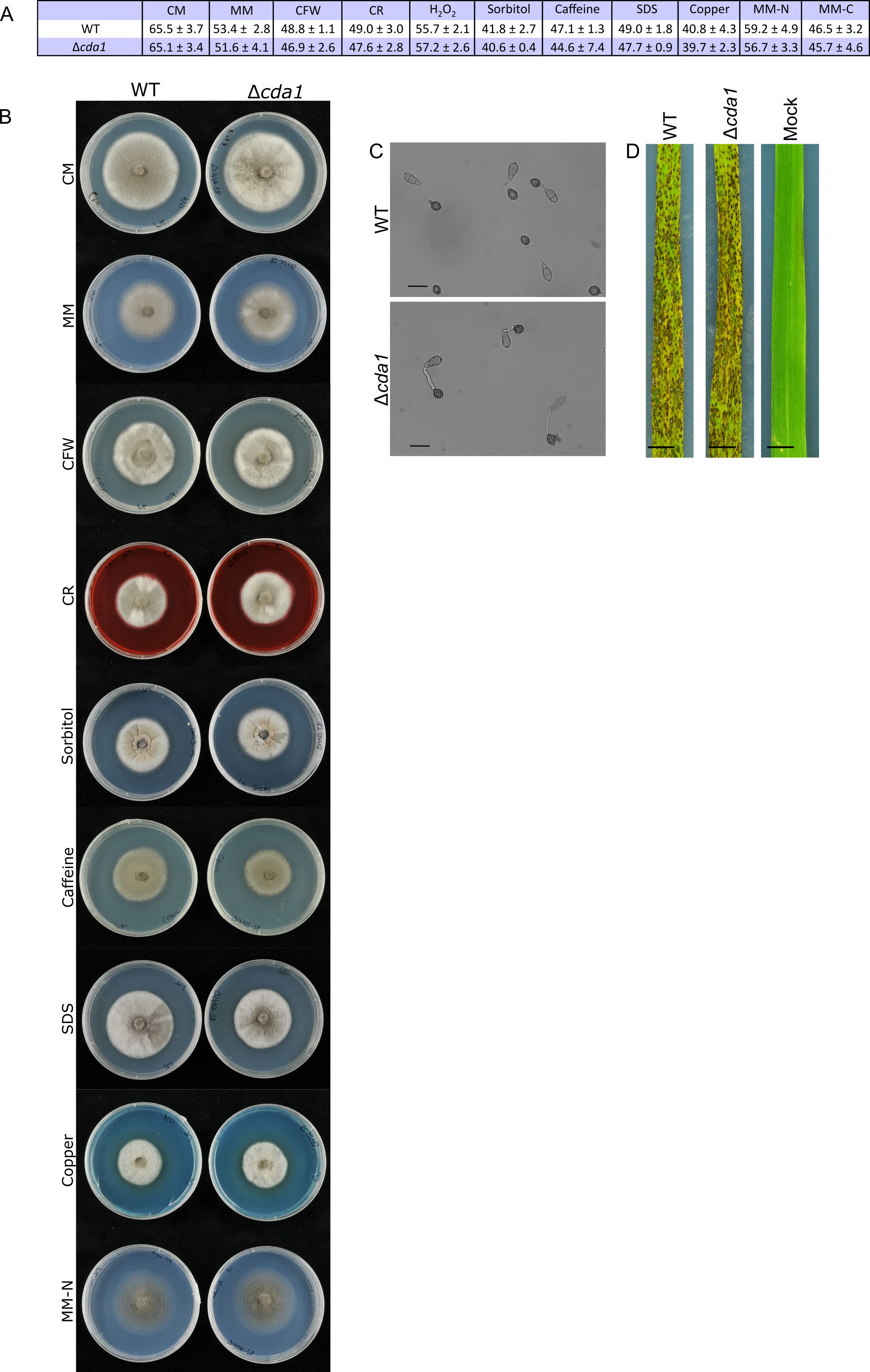

Supplement: Supplementary file 1 — Data S1. Figure S1. Antibody staining of chitosan in vegetative hyphae. A & B) Mycelial pellets of M.oryzae stained with the monoclonal anti‐chitosan antibody mAbG7. C) Secondary antibody only control, showing lack of staining. Scale bars: 20 μm. Figure S2. Domain architecture of CDA1, CDA4 and CDA5. CDA = Chitin deacetylase, CBD = Chitin binding domain. Figure S3. PCR analysis of CDA deletion strains. A) Schematic of targeted deletion strategy. Homologous recombination replaces the target gene with a gene imparting antibiotic resistance. B) PCR analysis of deletion strains. Putative deletion strains were screened by PCR to confirm the absence of the target gene (P1), and the integration of the deletion construct at the desired locus (P2 & P3). Position of primers shown in A. Figure S4. Southern Blot analysis of CDA deletion strains. Blots containing restriction digested gDNA of putative deletion strains were hybridised with α‐32P labelled DNA homologous to the hygromycin (HYG) (for CDA1 and CDA5) or bialaphos (BAR) (for CDA4) resistance genes. The cartoon above each blot shows the expected band size based upon the positions of the restriction enzymes sites at each locus. Size markers show band size in kilobases (kb). Successful single insertions were obtained for each of the 3 genes. In the ΔΔcda4/cda5 strain, cross‐hybridisation (band at ~20 kb) is observed between the HYG probe and the BAR gene used in the Δcda4 background strain. This is due to a common promoter sequence used in both the BAR and HYG resistance cassettes. Figure S5. Radial growth of Δcda1 strain under different stress conditions, and pathogenic development. A) Table of colony diameters (mm) (± SD, n = 3) of the WT and ∆cda1 strains grown on a range of different solid media, after 10 days incubation. B) Representative pictures of the Δcda1 strain growing on solid medium, taken after 10 days incubation. CM = Complete medium, MM = minimal medium, CFW = Calcofluor White, CR = Congo Red, SDS = Sodium [file CMI-19-na-s001.zip › FigureS5.tif]

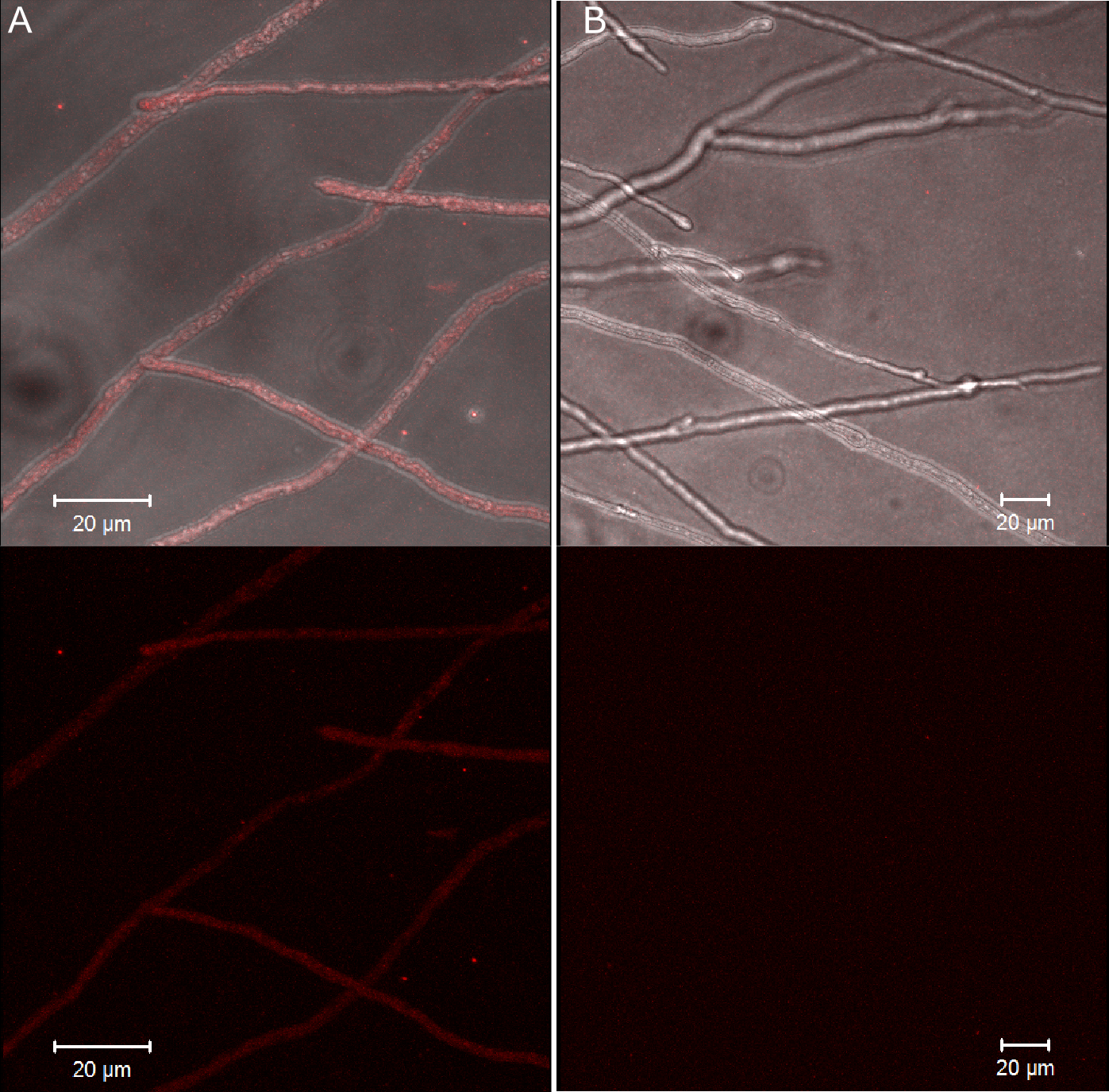

Supplement: Supplementary file 1 — Data S1. Figure S1. Antibody staining of chitosan in vegetative hyphae. A & B) Mycelial pellets of M.oryzae stained with the monoclonal anti‐chitosan antibody mAbG7. C) Secondary antibody only control, showing lack of staining. Scale bars: 20 μm. Figure S2. Domain architecture of CDA1, CDA4 and CDA5. CDA = Chitin deacetylase, CBD = Chitin binding domain. Figure S3. PCR analysis of CDA deletion strains. A) Schematic of targeted deletion strategy. Homologous recombination replaces the target gene with a gene imparting antibiotic resistance. B) PCR analysis of deletion strains. Putative deletion strains were screened by PCR to confirm the absence of the target gene (P1), and the integration of the deletion construct at the desired locus (P2 & P3). Position of primers shown in A. Figure S4. Southern Blot analysis of CDA deletion strains. Blots containing restriction digested gDNA of putative deletion strains were hybridised with α‐32P labelled DNA homologous to the hygromycin (HYG) (for CDA1 and CDA5) or bialaphos (BAR) (for CDA4) resistance genes. The cartoon above each blot shows the expected band size based upon the positions of the restriction enzymes sites at each locus. Size markers show band size in kilobases (kb). Successful single insertions were obtained for each of the 3 genes. In the ΔΔcda4/cda5 strain, cross‐hybridisation (band at ~20 kb) is observed between the HYG probe and the BAR gene used in the Δcda4 background strain. This is due to a common promoter sequence used in both the BAR and HYG resistance cassettes. Figure S5. Radial growth of Δcda1 strain under different stress conditions, and pathogenic development. A) Table of colony diameters (mm) (± SD, n = 3) of the WT and ∆cda1 strains grown on a range of different solid media, after 10 days incubation. B) Representative pictures of the Δcda1 strain growing on solid medium, taken after 10 days incubation. CM = Complete medium, MM = minimal medium, CFW = Calcofluor White, CR = Congo Red, SDS = Sodium [file CMI-19-na-s001.zip › FigureS6.tif]

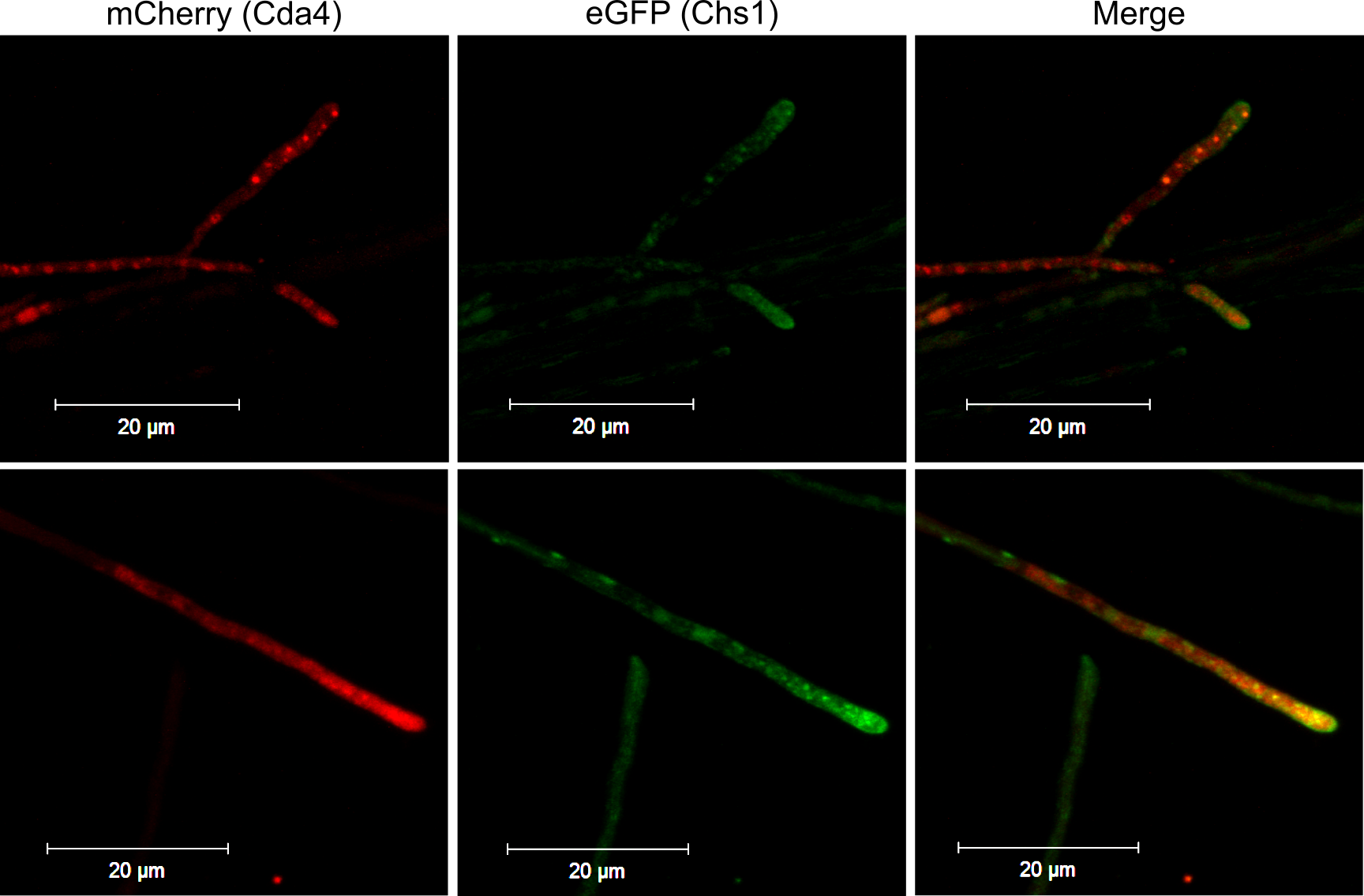

Supplement: Supplementary file 1 — Data S1. Figure S1. Antibody staining of chitosan in vegetative hyphae. A & B) Mycelial pellets of M.oryzae stained with the monoclonal anti‐chitosan antibody mAbG7. C) Secondary antibody only control, showing lack of staining. Scale bars: 20 μm. Figure S2. Domain architecture of CDA1, CDA4 and CDA5. CDA = Chitin deacetylase, CBD = Chitin binding domain. Figure S3. PCR analysis of CDA deletion strains. A) Schematic of targeted deletion strategy. Homologous recombination replaces the target gene with a gene imparting antibiotic resistance. B) PCR analysis of deletion strains. Putative deletion strains were screened by PCR to confirm the absence of the target gene (P1), and the integration of the deletion construct at the desired locus (P2 & P3). Position of primers shown in A. Figure S4. Southern Blot analysis of CDA deletion strains. Blots containing restriction digested gDNA of putative deletion strains were hybridised with α‐32P labelled DNA homologous to the hygromycin (HYG) (for CDA1 and CDA5) or bialaphos (BAR) (for CDA4) resistance genes. The cartoon above each blot shows the expected band size based upon the positions of the restriction enzymes sites at each locus. Size markers show band size in kilobases (kb). Successful single insertions were obtained for each of the 3 genes. In the ΔΔcda4/cda5 strain, cross‐hybridisation (band at ~20 kb) is observed between the HYG probe and the BAR gene used in the Δcda4 background strain. This is due to a common promoter sequence used in both the BAR and HYG resistance cassettes. Figure S5. Radial growth of Δcda1 strain under different stress conditions, and pathogenic development. A) Table of colony diameters (mm) (± SD, n = 3) of the WT and ∆cda1 strains grown on a range of different solid media, after 10 days incubation. B) Representative pictures of the Δcda1 strain growing on solid medium, taken after 10 days incubation. CM = Complete medium, MM = minimal medium, CFW = Calcofluor White, CR = Congo Red, SDS = Sodium [file CMI-19-na-s001.zip › FigureS7.tif]

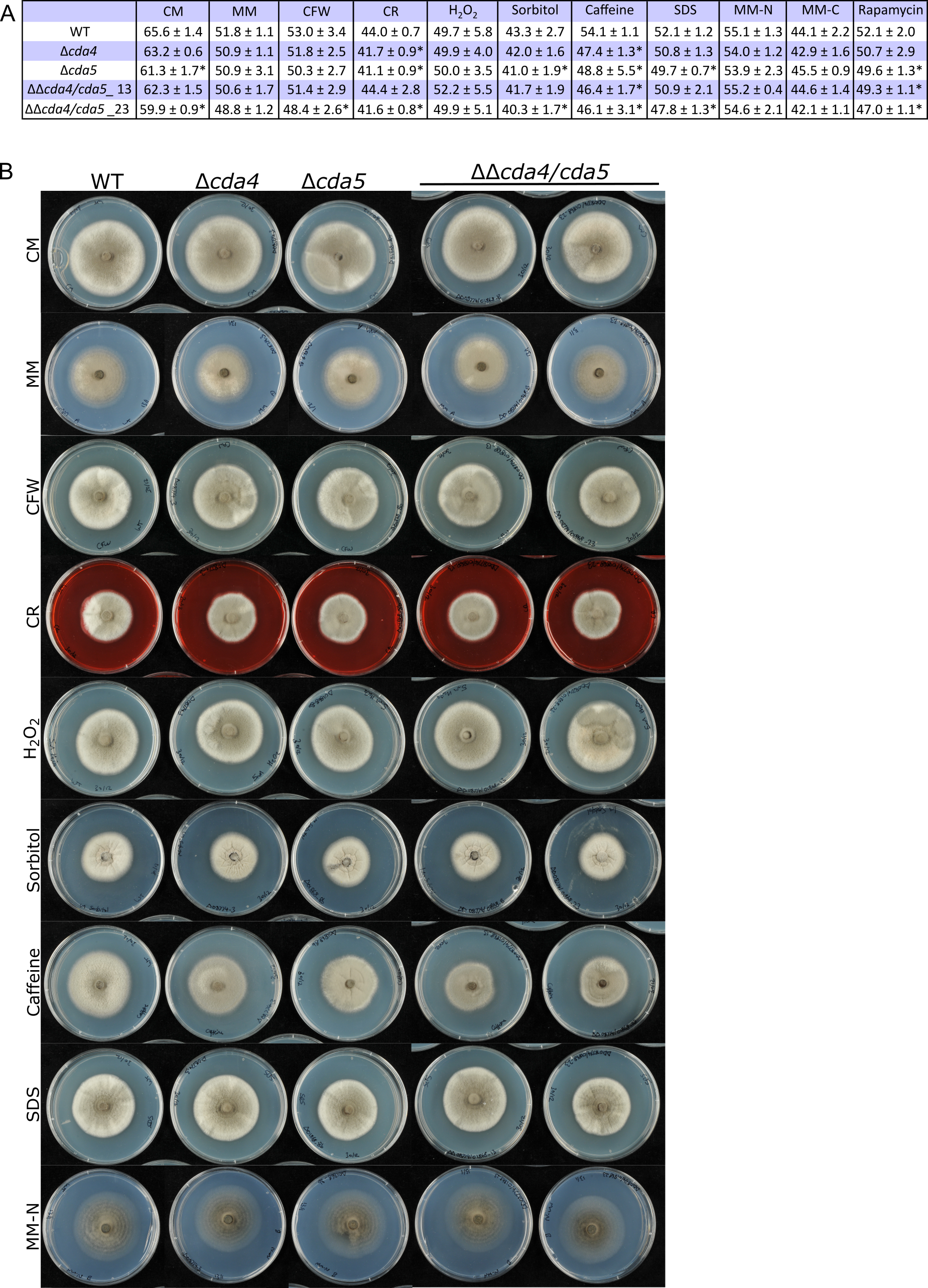

Supplement: Supplementary file 1 — Data S1. Figure S1. Antibody staining of chitosan in vegetative hyphae. A & B) Mycelial pellets of M.oryzae stained with the monoclonal anti‐chitosan antibody mAbG7. C) Secondary antibody only control, showing lack of staining. Scale bars: 20 μm. Figure S2. Domain architecture of CDA1, CDA4 and CDA5. CDA = Chitin deacetylase, CBD = Chitin binding domain. Figure S3. PCR analysis of CDA deletion strains. A) Schematic of targeted deletion strategy. Homologous recombination replaces the target gene with a gene imparting antibiotic resistance. B) PCR analysis of deletion strains. Putative deletion strains were screened by PCR to confirm the absence of the target gene (P1), and the integration of the deletion construct at the desired locus (P2 & P3). Position of primers shown in A. Figure S4. Southern Blot analysis of CDA deletion strains. Blots containing restriction digested gDNA of putative deletion strains were hybridised with α‐32P labelled DNA homologous to the hygromycin (HYG) (for CDA1 and CDA5) or bialaphos (BAR) (for CDA4) resistance genes. The cartoon above each blot shows the expected band size based upon the positions of the restriction enzymes sites at each locus. Size markers show band size in kilobases (kb). Successful single insertions were obtained for each of the 3 genes. In the ΔΔcda4/cda5 strain, cross‐hybridisation (band at ~20 kb) is observed between the HYG probe and the BAR gene used in the Δcda4 background strain. This is due to a common promoter sequence used in both the BAR and HYG resistance cassettes. Figure S5. Radial growth of Δcda1 strain under different stress conditions, and pathogenic development. A) Table of colony diameters (mm) (± SD, n = 3) of the WT and ∆cda1 strains grown on a range of different solid media, after 10 days incubation. B) Representative pictures of the Δcda1 strain growing on solid medium, taken after 10 days incubation. CM = Complete medium, MM = minimal medium, CFW = Calcofluor White, CR = Congo Red, SDS = Sodium [file CMI-19-na-s001.zip › FigureS8.tif]

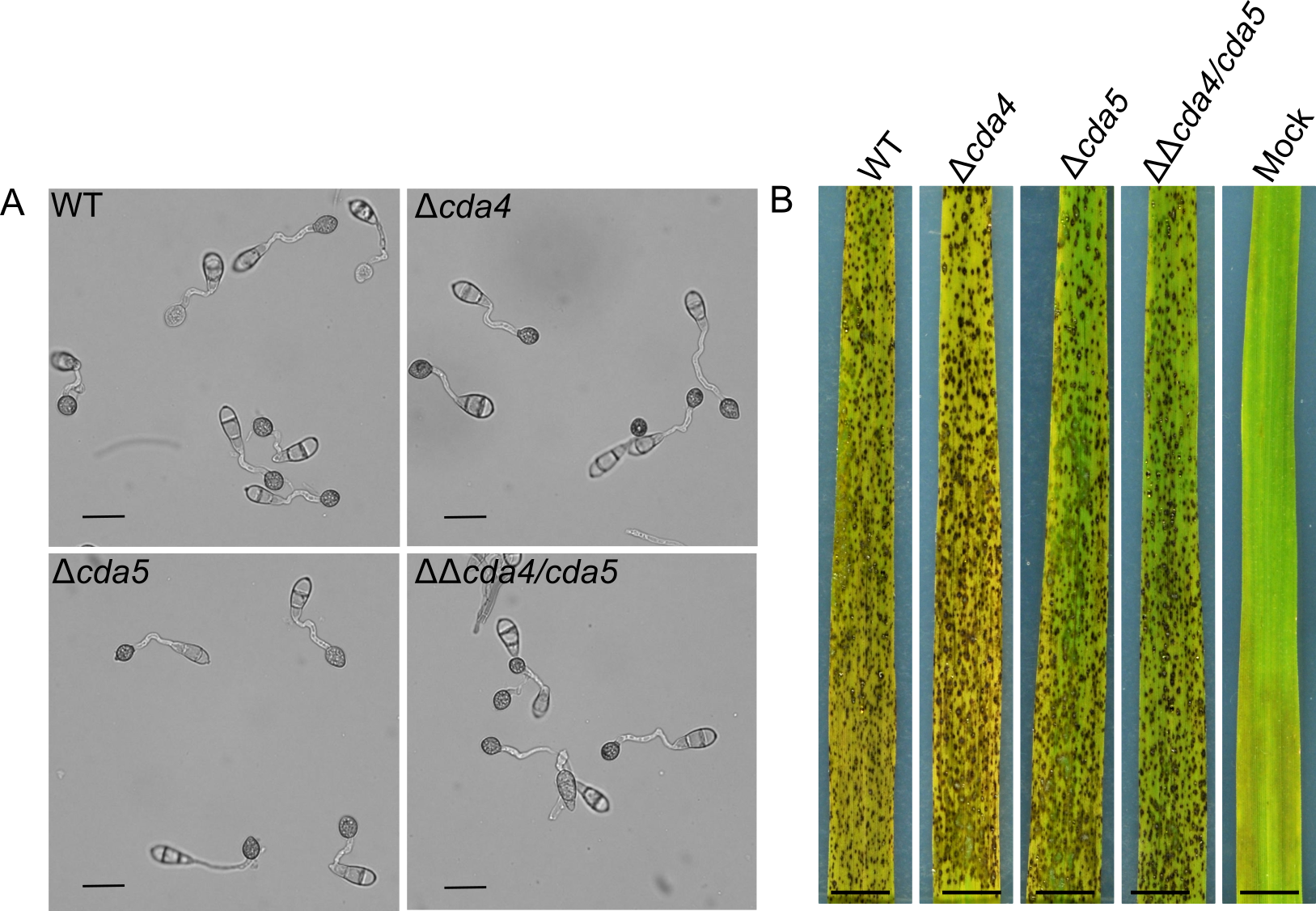

Supplement: Supplementary file 1 — Data S1. Figure S1. Antibody staining of chitosan in vegetative hyphae. A & B) Mycelial pellets of M.oryzae stained with the monoclonal anti‐chitosan antibody mAbG7. C) Secondary antibody only control, showing lack of staining. Scale bars: 20 μm. Figure S2. Domain architecture of CDA1, CDA4 and CDA5. CDA = Chitin deacetylase, CBD = Chitin binding domain. Figure S3. PCR analysis of CDA deletion strains. A) Schematic of targeted deletion strategy. Homologous recombination replaces the target gene with a gene imparting antibiotic resistance. B) PCR analysis of deletion strains. Putative deletion strains were screened by PCR to confirm the absence of the target gene (P1), and the integration of the deletion construct at the desired locus (P2 & P3). Position of primers shown in A. Figure S4. Southern Blot analysis of CDA deletion strains. Blots containing restriction digested gDNA of putative deletion strains were hybridised with α‐32P labelled DNA homologous to the hygromycin (HYG) (for CDA1 and CDA5) or bialaphos (BAR) (for CDA4) resistance genes. The cartoon above each blot shows the expected band size based upon the positions of the restriction enzymes sites at each locus. Size markers show band size in kilobases (kb). Successful single insertions were obtained for each of the 3 genes. In the ΔΔcda4/cda5 strain, cross‐hybridisation (band at ~20 kb) is observed between the HYG probe and the BAR gene used in the Δcda4 background strain. This is due to a common promoter sequence used in both the BAR and HYG resistance cassettes. Figure S5. Radial growth of Δcda1 strain under different stress conditions, and pathogenic development. A) Table of colony diameters (mm) (± SD, n = 3) of the WT and ∆cda1 strains grown on a range of different solid media, after 10 days incubation. B) Representative pictures of the Δcda1 strain growing on solid medium, taken after 10 days incubation. CM = Complete medium, MM = minimal medium, CFW = Calcofluor White, CR = Congo Red, SDS = Sodium [file CMI-19-na-s001.zip › FigureS9.tif]

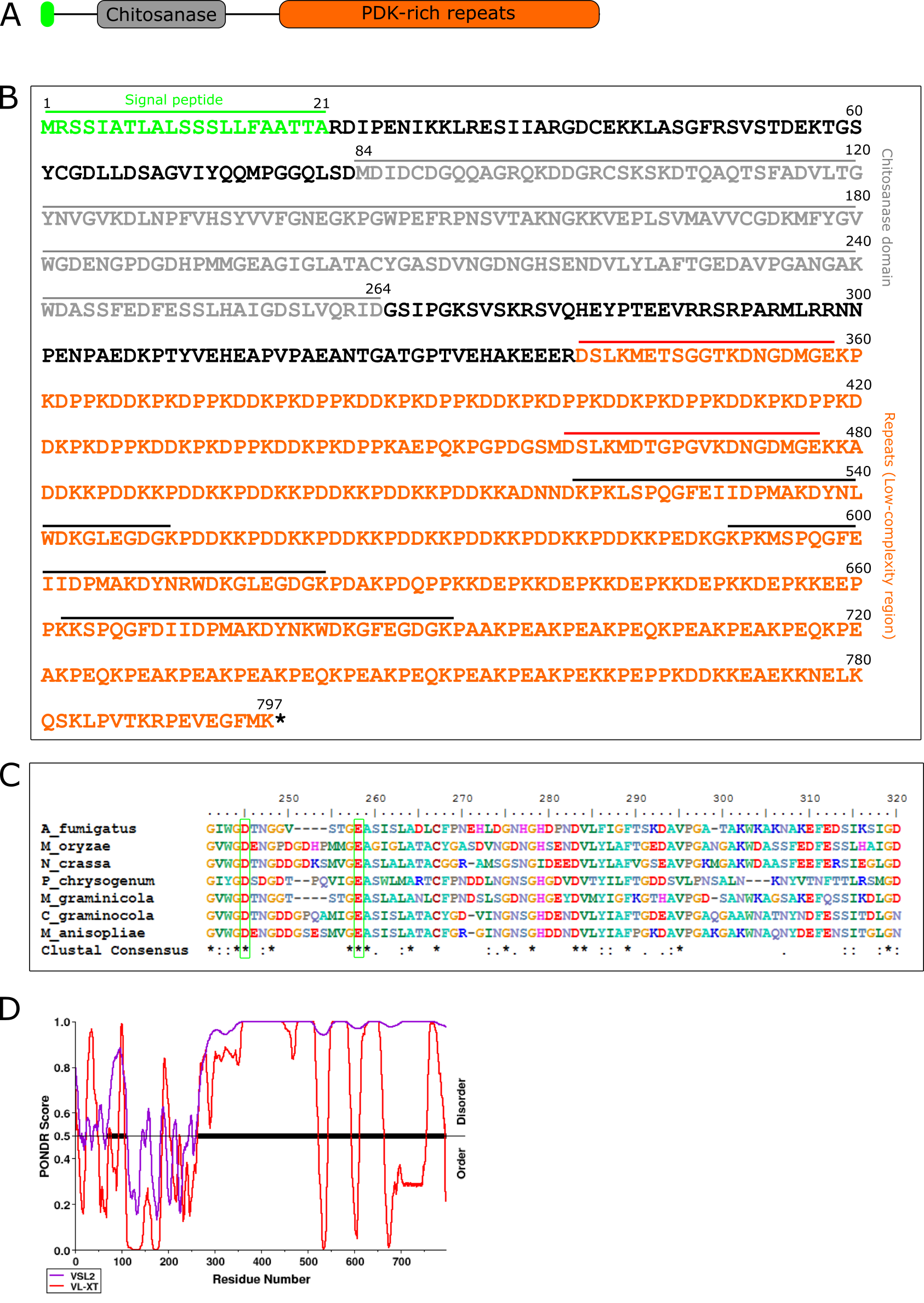

Supplement: Supplementary file 1 — Data S1. Figure S1. Antibody staining of chitosan in vegetative hyphae. A & B) Mycelial pellets of M.oryzae stained with the monoclonal anti‐chitosan antibody mAbG7. C) Secondary antibody only control, showing lack of staining. Scale bars: 20 μm. Figure S2. Domain architecture of CDA1, CDA4 and CDA5. CDA = Chitin deacetylase, CBD = Chitin binding domain. Figure S3. PCR analysis of CDA deletion strains. A) Schematic of targeted deletion strategy. Homologous recombination replaces the target gene with a gene imparting antibiotic resistance. B) PCR analysis of deletion strains. Putative deletion strains were screened by PCR to confirm the absence of the target gene (P1), and the integration of the deletion construct at the desired locus (P2 & P3). Position of primers shown in A. Figure S4. Southern Blot analysis of CDA deletion strains. Blots containing restriction digested gDNA of putative deletion strains were hybridised with α‐32P labelled DNA homologous to the hygromycin (HYG) (for CDA1 and CDA5) or bialaphos (BAR) (for CDA4) resistance genes. The cartoon above each blot shows the expected band size based upon the positions of the restriction enzymes sites at each locus. Size markers show band size in kilobases (kb). Successful single insertions were obtained for each of the 3 genes. In the ΔΔcda4/cda5 strain, cross‐hybridisation (band at ~20 kb) is observed between the HYG probe and the BAR gene used in the Δcda4 background strain. This is due to a common promoter sequence used in both the BAR and HYG resistance cassettes. Figure S5. Radial growth of Δcda1 strain under different stress conditions, and pathogenic development. A) Table of colony diameters (mm) (± SD, n = 3) of the WT and ∆cda1 strains grown on a range of different solid media, after 10 days incubation. B) Representative pictures of the Δcda1 strain growing on solid medium, taken after 10 days incubation. CM = Complete medium, MM = minimal medium, CFW = Calcofluor White, CR = Congo Red, SDS = Sodium [file CMI-19-na-s001.zip › FigureS10.tif]

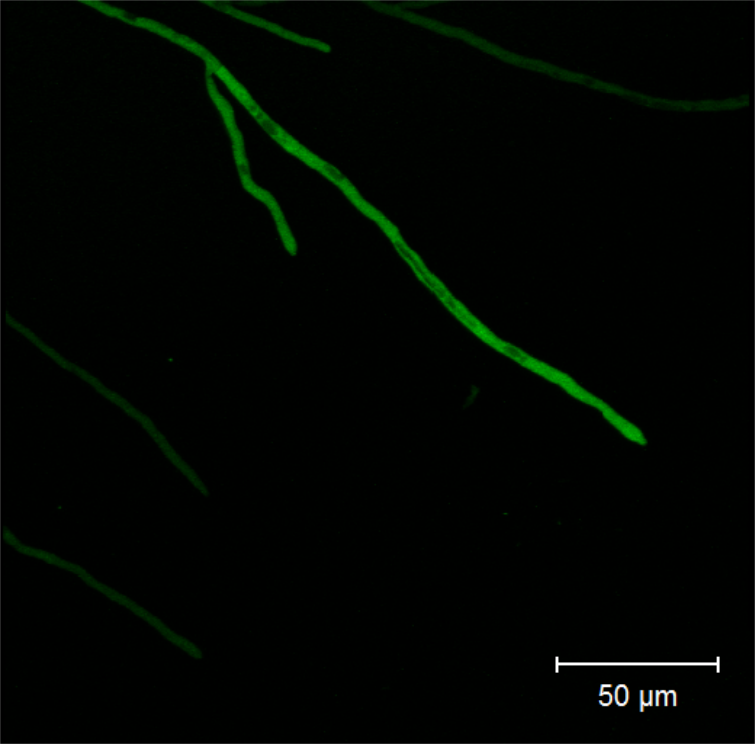

Supplement: Supplementary file 1 — Data S1. Figure S1. Antibody staining of chitosan in vegetative hyphae. A & B) Mycelial pellets of M.oryzae stained with the monoclonal anti‐chitosan antibody mAbG7. C) Secondary antibody only control, showing lack of staining. Scale bars: 20 μm. Figure S2. Domain architecture of CDA1, CDA4 and CDA5. CDA = Chitin deacetylase, CBD = Chitin binding domain. Figure S3. PCR analysis of CDA deletion strains. A) Schematic of targeted deletion strategy. Homologous recombination replaces the target gene with a gene imparting antibiotic resistance. B) PCR analysis of deletion strains. Putative deletion strains were screened by PCR to confirm the absence of the target gene (P1), and the integration of the deletion construct at the desired locus (P2 & P3). Position of primers shown in A. Figure S4. Southern Blot analysis of CDA deletion strains. Blots containing restriction digested gDNA of putative deletion strains were hybridised with α‐32P labelled DNA homologous to the hygromycin (HYG) (for CDA1 and CDA5) or bialaphos (BAR) (for CDA4) resistance genes. The cartoon above each blot shows the expected band size based upon the positions of the restriction enzymes sites at each locus. Size markers show band size in kilobases (kb). Successful single insertions were obtained for each of the 3 genes. In the ΔΔcda4/cda5 strain, cross‐hybridisation (band at ~20 kb) is observed between the HYG probe and the BAR gene used in the Δcda4 background strain. This is due to a common promoter sequence used in both the BAR and HYG resistance cassettes. Figure S5. Radial growth of Δcda1 strain under different stress conditions, and pathogenic development. A) Table of colony diameters (mm) (± SD, n = 3) of the WT and ∆cda1 strains grown on a range of different solid media, after 10 days incubation. B) Representative pictures of the Δcda1 strain growing on solid medium, taken after 10 days incubation. CM = Complete medium, MM = minimal medium, CFW = Calcofluor White, CR = Congo Red, SDS = Sodium [file CMI-19-na-s001.zip › FigureS11.tif]

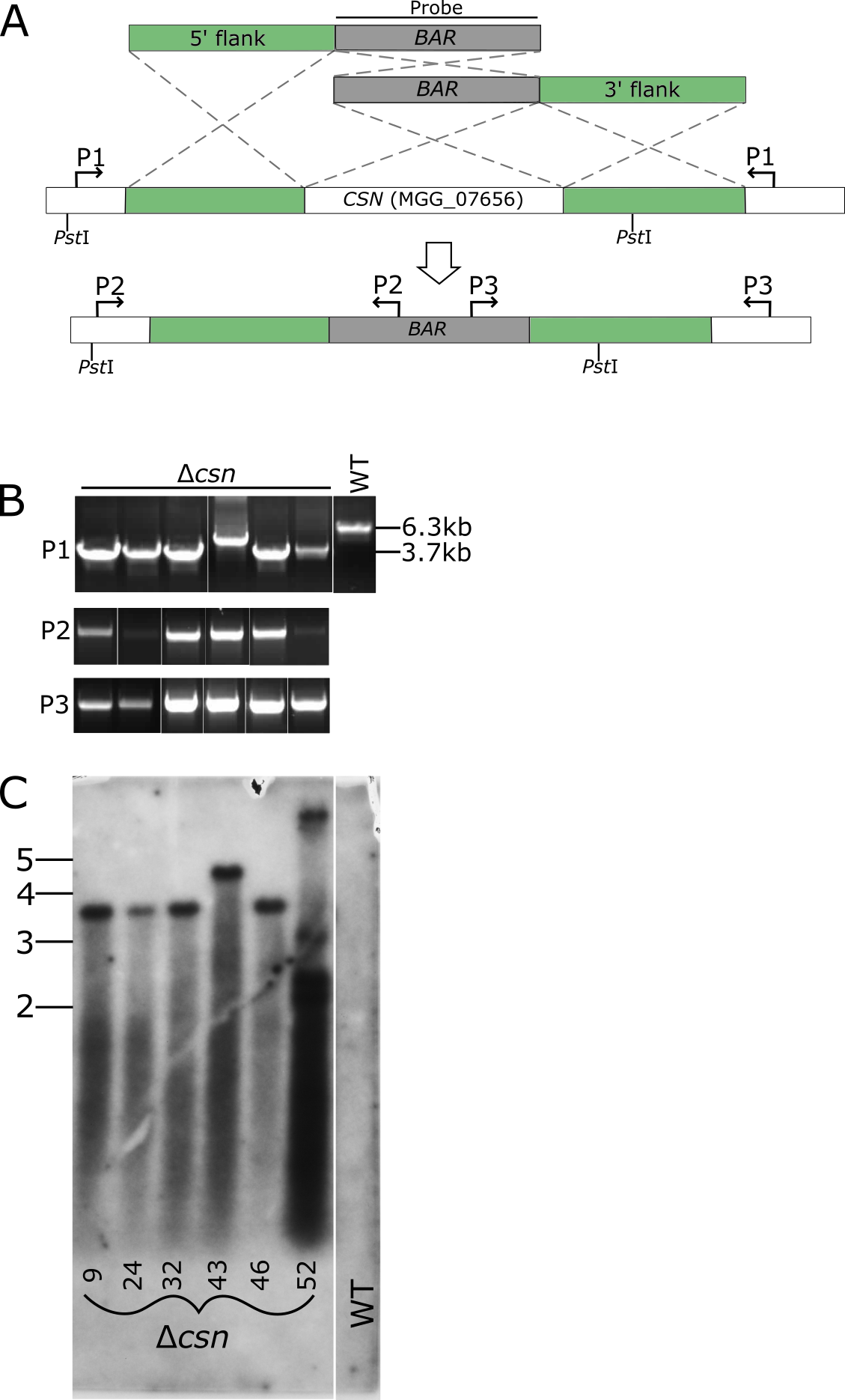

Supplement: Supplementary file 1 — Data S1. Figure S1. Antibody staining of chitosan in vegetative hyphae. A & B) Mycelial pellets of M.oryzae stained with the monoclonal anti‐chitosan antibody mAbG7. C) Secondary antibody only control, showing lack of staining. Scale bars: 20 μm. Figure S2. Domain architecture of CDA1, CDA4 and CDA5. CDA = Chitin deacetylase, CBD = Chitin binding domain. Figure S3. PCR analysis of CDA deletion strains. A) Schematic of targeted deletion strategy. Homologous recombination replaces the target gene with a gene imparting antibiotic resistance. B) PCR analysis of deletion strains. Putative deletion strains were screened by PCR to confirm the absence of the target gene (P1), and the integration of the deletion construct at the desired locus (P2 & P3). Position of primers shown in A. Figure S4. Southern Blot analysis of CDA deletion strains. Blots containing restriction digested gDNA of putative deletion strains were hybridised with α‐32P labelled DNA homologous to the hygromycin (HYG) (for CDA1 and CDA5) or bialaphos (BAR) (for CDA4) resistance genes. The cartoon above each blot shows the expected band size based upon the positions of the restriction enzymes sites at each locus. Size markers show band size in kilobases (kb). Successful single insertions were obtained for each of the 3 genes. In the ΔΔcda4/cda5 strain, cross‐hybridisation (band at ~20 kb) is observed between the HYG probe and the BAR gene used in the Δcda4 background strain. This is due to a common promoter sequence used in both the BAR and HYG resistance cassettes. Figure S5. Radial growth of Δcda1 strain under different stress conditions, and pathogenic development. A) Table of colony diameters (mm) (± SD, n = 3) of the WT and ∆cda1 strains grown on a range of different solid media, after 10 days incubation. B) Representative pictures of the Δcda1 strain growing on solid medium, taken after 10 days incubation. CM = Complete medium, MM = minimal medium, CFW = Calcofluor White, CR = Congo Red, SDS = Sodium [file CMI-19-na-s001.zip › FigureS12.tif]

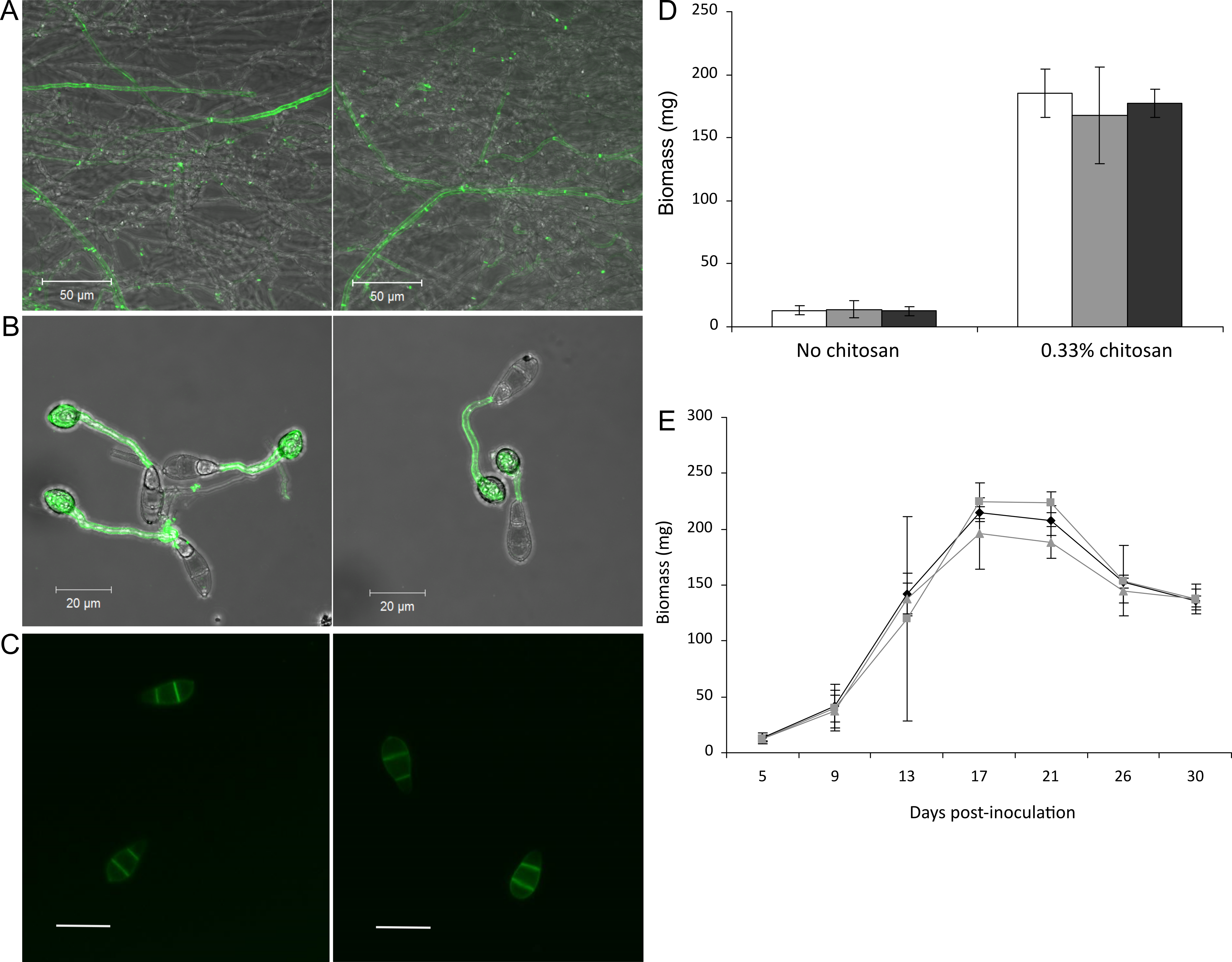

Supplement: Supplementary file 1 — Data S1. Figure S1. Antibody staining of chitosan in vegetative hyphae. A & B) Mycelial pellets of M.oryzae stained with the monoclonal anti‐chitosan antibody mAbG7. C) Secondary antibody only control, showing lack of staining. Scale bars: 20 μm. Figure S2. Domain architecture of CDA1, CDA4 and CDA5. CDA = Chitin deacetylase, CBD = Chitin binding domain. Figure S3. PCR analysis of CDA deletion strains. A) Schematic of targeted deletion strategy. Homologous recombination replaces the target gene with a gene imparting antibiotic resistance. B) PCR analysis of deletion strains. Putative deletion strains were screened by PCR to confirm the absence of the target gene (P1), and the integration of the deletion construct at the desired locus (P2 & P3). Position of primers shown in A. Figure S4. Southern Blot analysis of CDA deletion strains. Blots containing restriction digested gDNA of putative deletion strains were hybridised with α‐32P labelled DNA homologous to the hygromycin (HYG) (for CDA1 and CDA5) or bialaphos (BAR) (for CDA4) resistance genes. The cartoon above each blot shows the expected band size based upon the positions of the restriction enzymes sites at each locus. Size markers show band size in kilobases (kb). Successful single insertions were obtained for each of the 3 genes. In the ΔΔcda4/cda5 strain, cross‐hybridisation (band at ~20 kb) is observed between the HYG probe and the BAR gene used in the Δcda4 background strain. This is due to a common promoter sequence used in both the BAR and HYG resistance cassettes. Figure S5. Radial growth of Δcda1 strain under different stress conditions, and pathogenic development. A) Table of colony diameters (mm) (± SD, n = 3) of the WT and ∆cda1 strains grown on a range of different solid media, after 10 days incubation. B) Representative pictures of the Δcda1 strain growing on solid medium, taken after 10 days incubation. CM = Complete medium, MM = minimal medium, CFW = Calcofluor White, CR = Congo Red, SDS = Sodium [file CMI-19-na-s001.zip › FigureS13.tif]

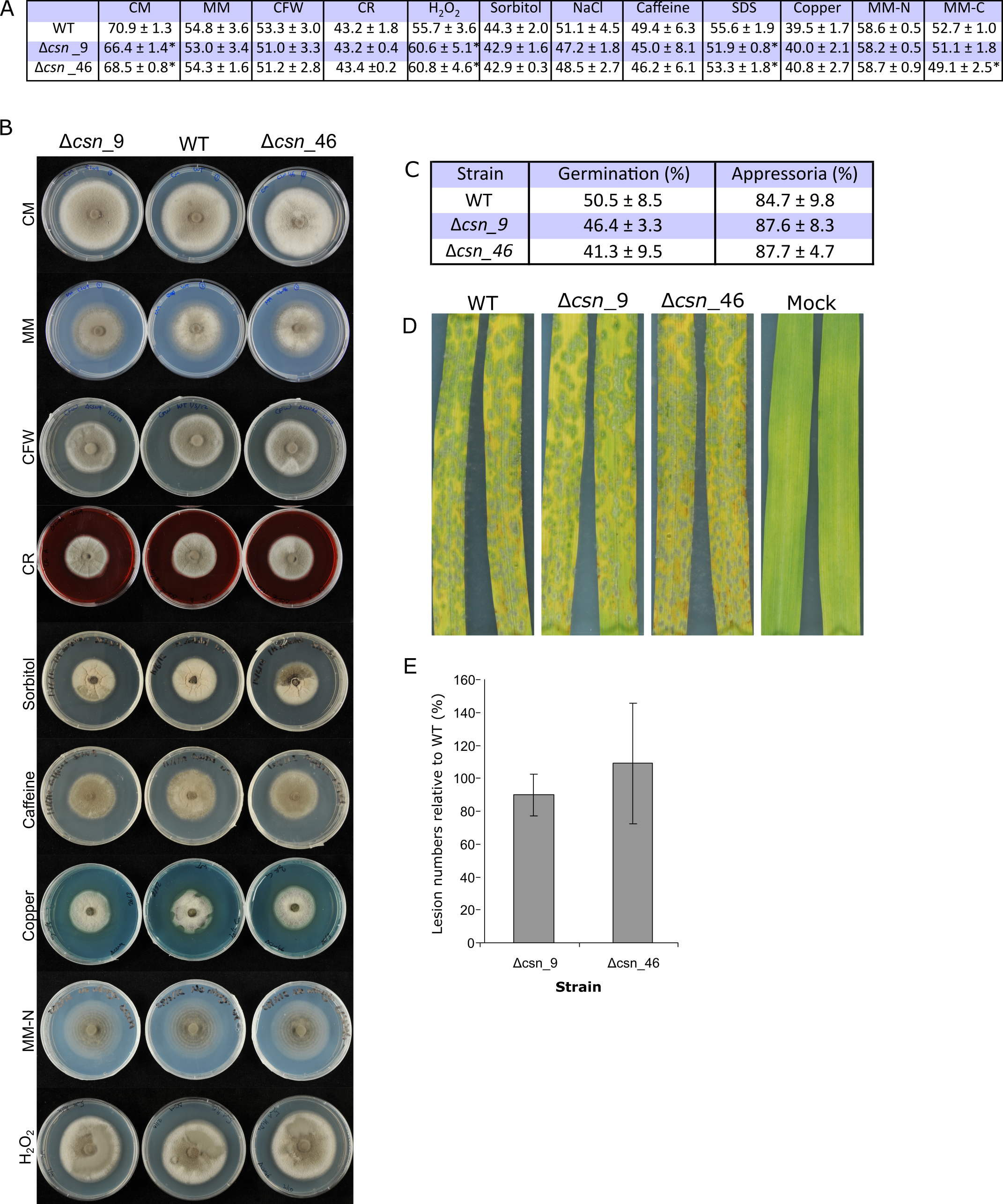

Supplement: Supplementary file 1 — Data S1. Figure S1. Antibody staining of chitosan in vegetative hyphae. A & B) Mycelial pellets of M.oryzae stained with the monoclonal anti‐chitosan antibody mAbG7. C) Secondary antibody only control, showing lack of staining. Scale bars: 20 μm. Figure S2. Domain architecture of CDA1, CDA4 and CDA5. CDA = Chitin deacetylase, CBD = Chitin binding domain. Figure S3. PCR analysis of CDA deletion strains. A) Schematic of targeted deletion strategy. Homologous recombination replaces the target gene with a gene imparting antibiotic resistance. B) PCR analysis of deletion strains. Putative deletion strains were screened by PCR to confirm the absence of the target gene (P1), and the integration of the deletion construct at the desired locus (P2 & P3). Position of primers shown in A. Figure S4. Southern Blot analysis of CDA deletion strains. Blots containing restriction digested gDNA of putative deletion strains were hybridised with α‐32P labelled DNA homologous to the hygromycin (HYG) (for CDA1 and CDA5) or bialaphos (BAR) (for CDA4) resistance genes. The cartoon above each blot shows the expected band size based upon the positions of the restriction enzymes sites at each locus. Size markers show band size in kilobases (kb). Successful single insertions were obtained for each of the 3 genes. In the ΔΔcda4/cda5 strain, cross‐hybridisation (band at ~20 kb) is observed between the HYG probe and the BAR gene used in the Δcda4 background strain. This is due to a common promoter sequence used in both the BAR and HYG resistance cassettes. Figure S5. Radial growth of Δcda1 strain under different stress conditions, and pathogenic development. A) Table of colony diameters (mm) (± SD, n = 3) of the WT and ∆cda1 strains grown on a range of different solid media, after 10 days incubation. B) Representative pictures of the Δcda1 strain growing on solid medium, taken after 10 days incubation. CM = Complete medium, MM = minimal medium, CFW = Calcofluor White, CR = Congo Red, SDS = Sodium [file CMI-19-na-s001.zip › FigureS14.tif]

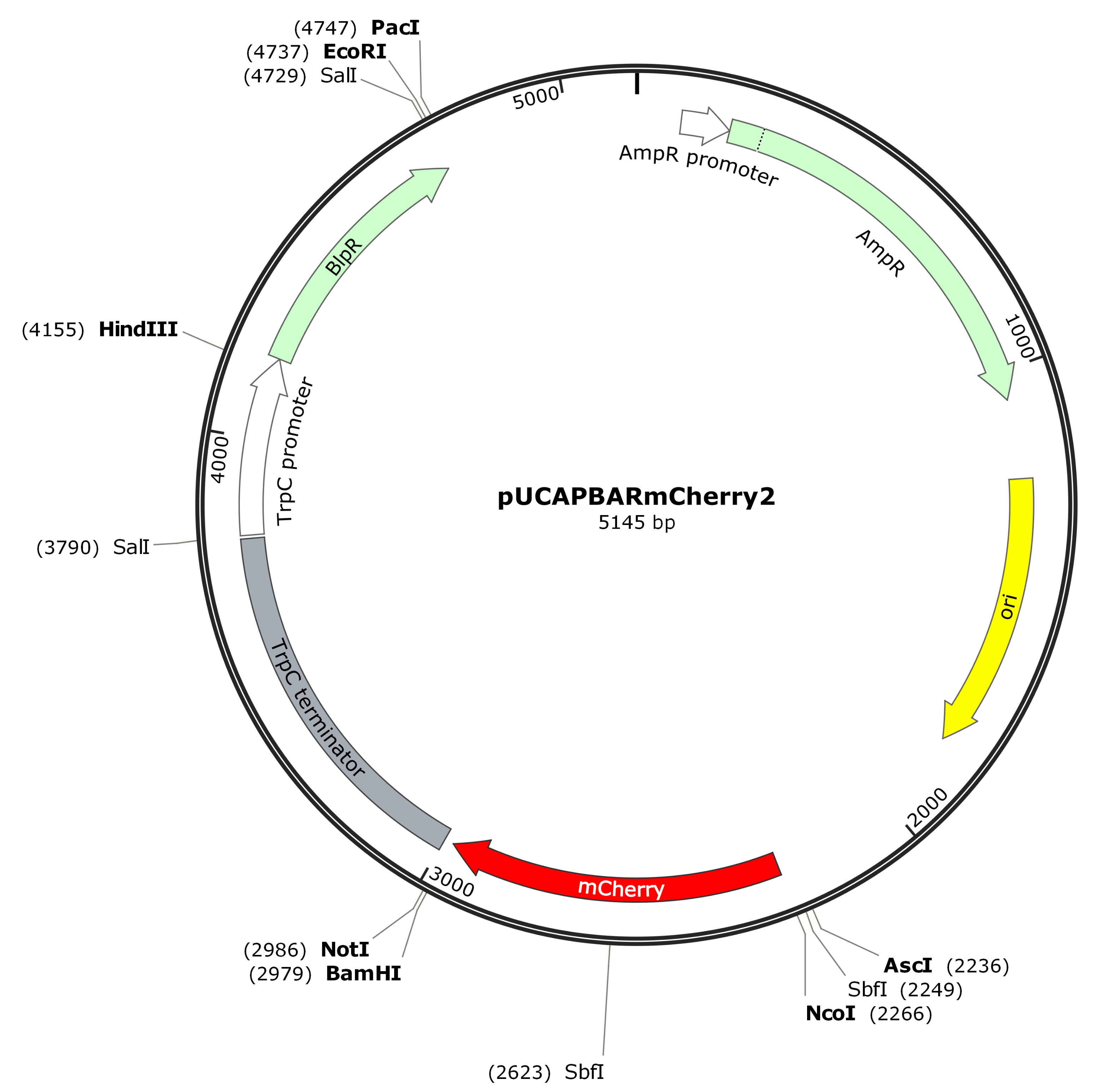

Supplement: Supplementary file 1 — Data S1. Figure S1. Antibody staining of chitosan in vegetative hyphae. A & B) Mycelial pellets of M.oryzae stained with the monoclonal anti‐chitosan antibody mAbG7. C) Secondary antibody only control, showing lack of staining. Scale bars: 20 μm. Figure S2. Domain architecture of CDA1, CDA4 and CDA5. CDA = Chitin deacetylase, CBD = Chitin binding domain. Figure S3. PCR analysis of CDA deletion strains. A) Schematic of targeted deletion strategy. Homologous recombination replaces the target gene with a gene imparting antibiotic resistance. B) PCR analysis of deletion strains. Putative deletion strains were screened by PCR to confirm the absence of the target gene (P1), and the integration of the deletion construct at the desired locus (P2 & P3). Position of primers shown in A. Figure S4. Southern Blot analysis of CDA deletion strains. Blots containing restriction digested gDNA of putative deletion strains were hybridised with α‐32P labelled DNA homologous to the hygromycin (HYG) (for CDA1 and CDA5) or bialaphos (BAR) (for CDA4) resistance genes. The cartoon above each blot shows the expected band size based upon the positions of the restriction enzymes sites at each locus. Size markers show band size in kilobases (kb). Successful single insertions were obtained for each of the 3 genes. In the ΔΔcda4/cda5 strain, cross‐hybridisation (band at ~20 kb) is observed between the HYG probe and the BAR gene used in the Δcda4 background strain. This is due to a common promoter sequence used in both the BAR and HYG resistance cassettes. Figure S5. Radial growth of Δcda1 strain under different stress conditions, and pathogenic development. A) Table of colony diameters (mm) (± SD, n = 3) of the WT and ∆cda1 strains grown on a range of different solid media, after 10 days incubation. B) Representative pictures of the Δcda1 strain growing on solid medium, taken after 10 days incubation. CM = Complete medium, MM = minimal medium, CFW = Calcofluor White, CR = Congo Red, SDS = Sodium [file CMI-19-na-s001.zip › FigureS15 plasmidmap.tif]
